# Why are patients dissatisfied following a total knee replacement? A systematic review

**DOI:** 10.1007/s00264-020-04607-9

**Published:** 2020-07-08

**Authors:** Naoki Nakano, Haitham Shoman, Fernando Olavarria, Tomoyuki Matsumoto, Ryosuke Kuroda, Vikas Khanduja

**Affiliations:** 1 Department of Trauma and Orthopaedics, Addenbrooke’s—Cambridge University Hospitals NHS Foundation Trust, Box 37, Hills Road, Cambridge, CB2 0QQ UK; 2 Department of Orthopaedic Surgery, Kobe University Graduate School of Medicine, 7-5-1 Kusunoki-cho, Chuo-ku, Kobe, 650-0017 Japan

**Keywords:** Total knee replacement, Total knee arthroplasty, Satisfaction, Dissatisfaction, Systematic review

## Abstract

**Background:**

Although total knee replacement (TKR) is an effective intervention for end-stage arthritis of the knee, a significant number of patients remain dissatisfied following this procedure. Our aim was to identify and assess the factors affecting patient satisfaction following a TKR.

**Materials and methods:**

In accordance with the PRISMA guidelines, two reviewers searched the online databases for literature describing factors affecting patient satisfaction following a TKR. The research question and eligibility criteria were established a priori. Any clinical outcome study that described factors relating to overall satisfaction after primary TKR was included. Quality assessment for the included studies was performed by two accredited orthopaedic surgeons experienced in clinical research.

**Results:**

The systematic review identified 181 relevant articles in total. A history of mental health problems was the most frequently reported factor affecting patient satisfaction (13 reportings). When the results of the quality assessment were taken into consideration, a negative history of mental health problems, use of a mobile-bearing insert, patellar resurfacing, severe pre-operative radiological degenerative change, negative history of low back pain, no/less post-operative pain, good post-operative physical function and pre-operative expectations being met were considered to be important factors leading to better patient satisfaction following a TKR.

**Conclusion:**

Surgeons performing a TKR should take these factors into consideration prior to deciding whether a patient is suitable for a TKR. Secondarily, a detailed explanation of these factors should form part of the process of informed consent to achieve better patient satisfaction following TKR. There is a great need for a unified approach to assessing satisfaction following a TKR and also the time at which satisfaction is assessed.

**Electronic supplementary material:**

The online version of this article (10.1007/s00264-020-04607-9) contains supplementary material, which is available to authorized users.

## Introduction

Total knee replacement (TKR) is one of the most effective surgical interventions for relief of pain and functional recovery in patients with advanced osteoarthritis (OA) of the knee. Management of OA costs the UK economy equivalent to 1% of its gross national product per year [1]. In the USA, the annual number of TKRs has been projected to rise by over 670% to 3.48 million cases by 2030 [2]. Outcomes of TKR are traditionally assessed by survival analysis with revision as the end point, and technical outcomes of this intervention are excellent. According to the UK National Joint Registry (NJR) annual report, the survival rate has been reported to be over 99.5% after one year and 95.6% at ten years [3].

A revision TKR is most commonly performed for loosening, fracture or infection. However, survival analysis tends to underestimate poor function, pain or dissatisfaction because these problems do not necessarily lead to a revision and are not recorded in the registry. Another issue is that reporting of the outcome of a TKR has predominantly been based on surgeon-derived outcome measures, which include range of movement (ROM), joint stability and post-operative alignment [4–6]. However, a report identified a poor correlation between surgeon-derived and patient-reported outcomes, with surgeons overestimating outcomes in comparison with the patients’ [7]. This correlates well with the fact that a significant number of patients experience continual pain and functional disability and therefore remain dissatisfied following the procedure [8–10].

In the largest ever reported series on satisfaction following a TKR, which included a survey of 27,372 patients, 17% of the unrevised patients were either dissatisfied or uncertain regarding their outcome [11]. Baker et al. [12] also reviewed the data from the NJR in the UK and reported that 71% of the patients experienced improvement of knee symptoms, but only 22% of them rated the results as excellent. Therefore, although the surgeon-reported outcomes may be good and the patient has no indication for a revision, they may still be dissatisfied following their index TKR. This may be due to a multitude of reasons, but to the best of our knowledge, there has been no systematic review which has specifically focused on the factors that affect patient satisfaction following a TKR. The aim of this systematic review, therefore, was to identify and assess the factors affecting patient satisfaction following a TKR.

## Methods

The protocol of this systematic review was developed and has been registered in the International Prospective Register of Systematic Reviews (PROSPERO 2017 CRD42017084659). The Preferred Reporting Items for Systematic Reviews and Meta-Analyses (PRISMA) guidelines were used for designing this study [13].

### Search strategy

Two accredited orthopaedic surgeons experienced in clinical research searched the online database Medline, Embase, BNI, AMED, Cochrane and Google Scholar for literature relating to satisfaction following a TKR. The PICO (participants, interventions, comparators, outcomes) tool was adopted and modified to formulate the research question and establish the inclusion and exclusion criteria. Selected articles were then exported to Mendeley reference manager software to organise screen and select articles.

### Study screening and selection

Clinical outcome studies that described the factors relating to the overall or general satisfaction/dissatisfaction following a primary TKR irrespective of any pathology were included. The inclusion and exclusion criteria are described in Table 1. Any discrepancies at the title and abstract revision stage were resolved by automatic inclusion to ensure thoroughness. Any discrepancies at the full-text stage were resolved by consensus between the two reviewers. If a consensus could not be reached, a third, more senior reviewer was consulted to resolve the discrepancy.

**Table 1:** Inclusion and exclusion criteria applied to articles identified in the literature

Inclusion criteria	
1. All levels of evidence	
2. Written in the English language	
3. Studies on humans	
4. Studies reporting factors affecting overall satisfaction and/or dissatisfaction following a primary total knee replacement	
5. Operative procedure consisted solely of total knee replacement	
6. Total knee replacement irrespective of any pathology	
Exclusion criteria	
1. Studies whose results included other procedures	
2. Studies reporting satisfaction/dissatisfaction for only a small part of the procedure (e.g. ‘satisfaction in either pain control, skin closure, range of motion, nursing quality, anaesthesia, nerve block or physiotherapy’ was excluded)	
3. Studies not reporting patient’s satisfaction (e.g. ‘studies on family’s or carer’s satisfaction’ were excluded)	
4. Studies describing trial protocols without any results	
5. Studies with follow-up period of 3 months or less	
6. Revision total knee replacement	
7. Unicompartmental knee replacement	
8. Patellofemoral knee replacement	
9. Cadaveric or radiological studies	
10. Reviews, systematic reviews	

### Data extraction and analysis

The two reviewers independently extracted relevant study data from the final pool of included articles and recorded this data on a spreadsheet designed a priori in Microsoft Excel 2013 (Microsoft Corporation, Redmond, WA, USA). The quality of studies including bias was then analysed and assessed using the Joanna Briggs Institute Critical Appraisal Checklist (JBICAC) for cohort studies, case–control studies, cross-sectional studies and case series [14]. For RCTs, a modified version of critical appraisal checklist by van Tulder et al. was used [15].

### Statistical methods

Statistical analysis in this study focused on descriptive statistics. After assessing the quality of each study, the score was converted into a percentage from the full score (%), which was then considered to be the ‘strength’ of that particular study. Microsoft Excel 2013 was used for our analysis in reporting the factors affecting patient satisfaction following a TKR, based on the strength of studies as per the type of evidence. The potential factors were then categorised into seven groups designed from the findings of the studies included. The strength of each factor was presented, regardless of whether it was a FACTOR (‘it is a factor for patient satisfaction’) or a Not-FACTOR (‘it is a factor which does NOT relate to patient satisfaction’—in other words, ‘researcher X found Factor Z was irrelevant to patient satisfaction’).

Details are described in Electronic Supplementary Material 1 and Table 2.

**Table 2:** Search strategy for Medline

No.	Searches	Medline results
1	satisf$.mp. [mp=title, abstract, original title, name of substance word, subject heading word, keyword heading word, protocol supplementary concept word, rare disease supplementary concept word, unique identifier, synonyms]	366,508
2	tkr.mp. [mp=title, abstract, original title, name of substance word, subject heading word, keyword heading word, protocol supplementary concept word, rare disease supplementary concept word, unique identifier, synonyms]	1908
3	tka.mp. [mp=title, abstract, original title, name of substance word, subject heading word, keyword heading word, protocol supplementary concept word, rare disease supplementary concept word, unique identifier, synonyms]	8888
4	“total knee arthroplasty”.mp. [mp=title, abstract, original title, name of substance word, subject heading word, keyword heading word, protocol supplementary concept word, rare disease supplementary concept word, unique identifier, synonyms]	15,890
5	“total knee replacement”.mp. [mp=title, abstract, original title, name of substance word, subject heading word, keyword heading word, protocol supplementary concept word, rare disease supplementary concept word, unique identifier, synonyms]	5129
6	2 or 3 or 4 or 5	21,446
7	dissatisf$.mp. [mp=title, abstract, original title, name of substance word, subject heading word, keyword heading word, protocol supplementary concept word, rare disease supplementary concept word, unique identifier, synonyms]	17,906
8	1 or 7	374,612
9	6 and 8	2187

## Results

**Fig. 1:**
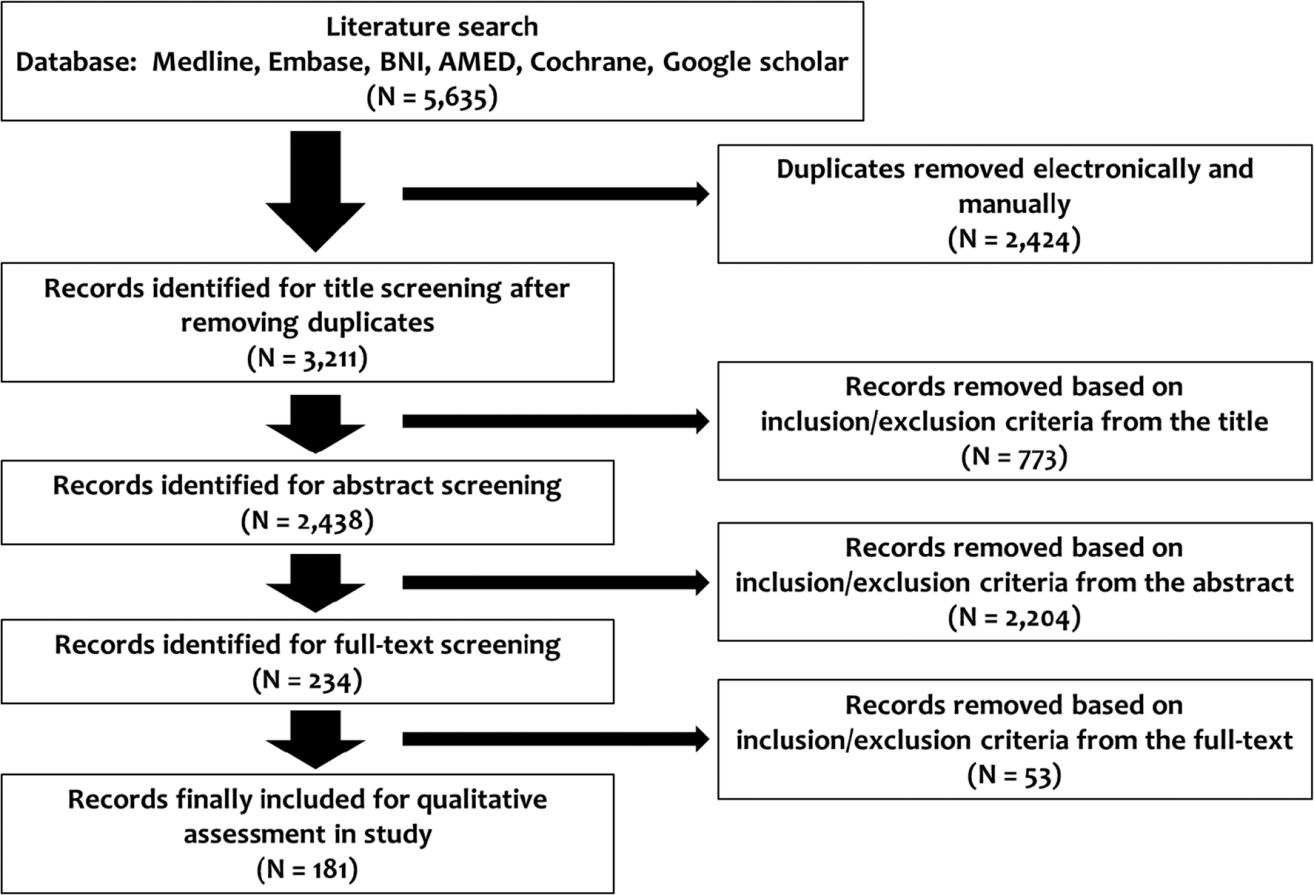
PRISMA flowchart for results of the literature database search

A total of 5635 articles were found following the initial search of the electronic databases and citation tracking, followed by removing 2424 duplicate articles. After review by title and abstract, 2977 articles were excluded and 234 potential articles remained for a full-text review. After application of the inclusion and exclusion criteria, a further 53 articles were discarded, leaving 181 relevant articles for the final inclusion, analysis and assessment. The study finally included 40 RCTs (22.1%), 93 cohort studies (51.4%), nine case–control studies (5.0%), 37 cross-sectional studies (20.4%) and 2 case series (1.1%) (Electronic Supplementary Material 2). Flowchart for the review is shown in Fig. 1 and the details of all the 181 studies are shown in Table 3. A total of 22 authors were found to have written several papers. To ensure that duplicate numbers were not included in our analysis, we contacted all these authors and reminder emails were sent as well to ensure a reply. Only five authors replied back with no overlap in their studies, three authors said that there was an overlap and 14 did not reply back. Those who did not reply back were treated as if it was an overlap and, thus, not considered. Due to the lack of homogeneity between studies, a meta-analysis was deemed unsuitable for this study.

**Table 3:** Details of 181 included studies (alphabetical order of the first author’s name)

First author	Serial no.	Factors affecting or relating to satisfaction	Measuring method for satisfaction	Year	Country	Type of study	Assessment timing	Number of TKRs	Men	Women	Age	BMI	Primary diagnosis	Surgical approach	Patellar resurface	Prosthesis	Use of cement	Anaesthesia
Adam	1	No difference between age 75 years or older and younger than 75 years	British Orthopaedic Association grading system	1994	UK	Cohort	Minimum 2 years	125	18	67	78 G1 64 G2	NA	OA	NA	NA	NA	NA	NA
Albayrak	2	Patient satisfaction was higher in patients with low pain intensity	4 grades (very satisfied, satisfied, dissatisfied, very dissatisfied)	2016	Turkey	Cross-sectional	22.8 months	274	NA	NA	66.8	32.3	OA	MPP	NA	NA	Yes	Spinal or combined (spinal + epidural)
Ali	3	(1) Very satisfied group had less pain, less anxiety or depression (2) Mean range of motion was 11 degrees greater in very satisfied group than the dissatisfied group	4 grades (very satisfied, satisfied, uncertain, dissatisfied)	2014	Sweden	Cross-sectional	10.5 years	118	32	82	78.5	31.0	OA	NA	NA	NA	NA	NA
Ali	4	No difference between patellar resurface group and non-resurface group	4 grades (very satisfied, satisfied, uncertain, dissatisfied)	2016	Sweden	Randomised controlled trial	6 years	74	29	45	68.5	30.0	OA	MPP	Yes and no	Triathlon (CR)	Yes	62 spinal, 12 general
Ali	5	(1) Patients with pre-operative anxiety or depression had more than 6 times higher risk to be dissatisfied compared with patients with no anxiety or depression (*P* < 0.001) (2) Patients with deep prosthetic infection had 3 times higher risk to be dissatisfied with the operation outcome (*P* < 0.03) (3) Dissatisfied patients had 1-day longer hospital stay compared with the satisfied patients (*P* < 0.001)	4 grades (very satisfied, satisfied, uncertain, dissatisfied)	2016	Sweden	Cohort	4 years	186	66	120	72.5	30.0	OA	MPP	NA	Triathlon (CR), PFC (CR)	NA	87% spinal, 13% general
Altay	6	No difference between midvastus approach and MPP	6 grades	2011	Turkey	Cohort	41.3 months	104	14	38	67.8	31.2	NA	MPP, midvastus	NA	Maxim (fixed bearing) (PS)	NA	Spinal
Anderson	7	(1) Poor mental health score, decreased physical function and increased bodily pain score negatively related to satisfaction (2) Age, gender, diagnosis, weight and pre-operative medical comorbidities did not relate to satisfaction	5 grades (very satisfied, somewhat satisfied, neutral, somewhat dissatisfied, very dissatisfied)	1996	USA	Cohort	2.85 years	119	33	86	79.6	NA	OA, RA	NA	NA	NA	NA	NA
Aunan	8	No difference between patellar resurfacing and non-resurfacing	VAS (0–100)	2016	Norway	Randomised controlled trial	3 years	129	48	67	70.0	29.5	OA	MPP	Yes and no	NexGen (fixed bearing) (CR)	Yes	NA
Baker	9	(1) Pain, women gender, OA, age younger than 65 and ASA 1 negatively related to satisfaction (2) Grade of the surgeon (consultant or not), site of the incision, use of a tourniquet and removal of the fat pad did not relate to satisfaction	3 grades (yes (satisfied), not sure, no)	2007	UK	Cross-sectional	Minimum 1 year	8231	3557	4671	70.8	NA	OA, other	NA	NA	NA	NA	NA
Baker	10	Patients with BMI > 35 were less satisfied than the control group (18.5 < BMI < 24)	4 grades (very satisfied, somewhat satisfied, somewhat dissatisfied, very dissatisfied)	2013	UK	Cohort	3 years	1367	585	782	68.8	29.5	OA	NA	NA	PFC, triathlon	Yes	NA
Baker	11	The perception of symptom improvement (operative success) positively related to satisfaction	5 grades (excellent, very good, good, fair, poor)	2013	UK	Cohort	199 days	22278	NA	NA	NA	NA	OA	NA	NA	NA	NA	NA
Barlow	12	No difference between (1) stemmed prosthesis and non-stemmed prosthesis; (2) short (< 80 mm) stem and long (> 80 mm) stem; (3) one stem and two stems	Satisfied or not	2016	USA	Cohort	2 years	13825	4977	8848	67.5	30.4	OA, inflammatory disease, AVN, post-trauma OA, fracture, other	NA	NA	NA	NA	NA
Barrack	13	No difference between patients with patellar resurfacing and those without it	Satisfied or not	2001	USA	Randomised controlled Trial	70.5 months	93	NA	NA	NA	NA	OA	NA	Yes and no	MG II (CR)	Yes	NA
Barrack	14	(1) Patients with incomes of less than USD 25,000, and women were less satisfied (2) Race, education, employment status and implant type (CR or PS, rotating platform, high flexion, gender specific) had no effect on satisfaction	Satisfied or not	2013	USA	Cohort	2.6 years	661	256	405	54.0	NA	OA	NA	NA	Unknown (52% CR, 27% PS, 9% rotating-platform, 6% high-flexion, 5% sex-specific)	NA	NA
Bican	15	Patients with fibromyalgia were less satisfied	4 grades (very satisfied, satisfied, dissatisfied, very dissatisfied)	2011	USA	Case–control	3.4 years	180	2	57	61.0	34.0	Fibromyalgia, OA	MPP	Yes	NA	Yes	Combined (spinal + epidural) or general
Bierke	16	Mean dissatisfaction scores were significantly higher in patients with somatisation dysfunction	5 grades (very satisfied, satisfied, mediocre satisfied, unsatisfied, very unsatisfied)	2016	Germany	Cohort	12 months	100	37	63	60.6	29.9	OA	MPP	No	Genesis II (CR)	Yes	General
Bierke	17	Patients with anxiety and particularly patients with pain catastrophizing tended to be dissatisfied	5 grades (very satisfied, satisfied, mediocrely satisfied, unsatisfied, very unsatisfied)	2017	Germany	Cohort	9 months	138	87	51	69.0	29.9	OA	MPP	NA	Genesis II	NA	General
Biyani	18	No difference between CS and PS	5 grades (very satisfied, satisfied, neutral, dissatisfied, very dissatisfied)	2017	USA	Cohort	1 year	82	0	82	66.5 (Median)	29.4 (Median)	NA	MPP	Yes	Triathlon (CS, PS)	NA	NA
Blyth	19	Using iNav Electromagnetic navigation system had no effect on satisfaction	6 grades	2015	UK	Randomised controlled trial	1 year	198	116	82	65.5	NA	OA	NA	NA	NexGen LPS-flex (PS)	Yes	NA
Boese	20	No difference between PFC Sigma rotating platform high flex and PFC Sigma rotating plat form	5 grades	2011	USA	Case–control	16.7 months	153	63	90	64.0	NA	OA	MPP	NA	PFC Sigma RP (rotating platform) (CR), PFC Sigma RP-F (rotating platform) (PS)	Yes	NA
Bonnin	21	Of the patients who reported they were as active as they expected to be before TKR, 98.2% were satisfied, while of the patients who reported they were insufficiently active, 52.3% were not satisfied (*P* < 0.0001)	5 grades (very satisfied, satisfied, moderately satisfied, somewhat dissatisfied, dissatisfied)	2010	France	Cross-sectional	44 months	347	120	227	75.0	27.9	OA, RA, AVN	NA	Yes and no	Noetos (PS), NexGen (PS)—282 mobile bearing, 65 fixed bearing	Cemented tibia 338 Cemented femur 337	NA
Bourne	22	Patients with expectations not met, pre-operative pain at rest, and a post-operative complication requiring hospital readmission were less satisfied	5 grades (very dissatisfied, dissatisfied, neutral, satisfied, very satisfied)	2010	Canada	Cross-sectional	1 year	1703	644	1059	69.3	32.0	OA, RA, post-trauma OA, other	NA	Yes and no	Unknown (53% CR, 47% PS)	NA	NA
Bugada	23	Higher BMI and anxiety/depression levels were associated with dissatisfaction	VAS (0–10)	2017	Italy	Cohort	6 months	563	185	421	72	NA	NA	NA	NA	NA	NA	General
Bullens	24	RA patients were more satisfied than OA patients	VAS (0–100)	2001	Netherlands	Cross-sectional	4.9 years	126	NA	NA	67.4	NA	OA, RA, juvenile rheumatoid arthritis, haemophilic arthropathy	NA	Yes and no	PFC (95% CR, 5% PS)	Yes	NA
Burnett	25	No difference between patients with patellar resurfacing and those without it	Original questionnaire (41 points)	2009	USA	Randomised controlled trial	Minimum 10 years	78	NA	NA	78.0	NA	NA	NA	Yes and no	MG II (CR)	Yes	NA
Burnett	26	No difference between patients with patellar resurfacing and those without it	Original questionnaire	2004	USA	Randomised controlled trial	7.3 years	90	39	51	70.0	31.9	OA	MPP	Yes and no	AMK (CR)	Hybrid (cemented tibia)	NA
Burnett	27	No difference between patients with patellar resurfacing and those without it	Original questionnaire (41 questions)	2007	USA	Randomised controlled trial	110 months	56	19	9	78.0	NA	OA	NA	Yes and no	MG II (fixed bearing) (CR)	Yes	General
Chang	28	Patients with regular physical activity after TKR were more satisfied	VAS (0–10)	2014	South Korea	Cohort	24 months	369	30	339	68.8	27.4	OA	NA	NA	NA	NA	NA
Chang	29	Post-operative severe pain relates to dissatisfaction	4 grades (enthusiastic, satisfied, noncommittal, disappointed)	2010	South Korea	Cross-sectional	1 year	383	10	230	68.8	26.2	OA	MPP	Yes	E-motion (mobile), Genesis II (fixed)	Yes	NA
Chinnappa	30	Radiologic leg length discrepancy (LLD) did not relate to patient satisfaction, but perception of LLD related to satisfaction	5 grades	2017	Australia	Cohort	6 months	91	34	57	70.2	29.4	OA, post-traumatic arthritis, AVN, RA	MPP	NA	PS implant made by Smith and Nephew	Yes	NA
Choi	31	No difference between standard PS rotating platform mobile bearing TKR and high flexion PS rotating platform mobile bearing TKR	5 grades	2010	South Korea	Randomised controlled trial	28 months	170	9	119	70.5	26.6	OA	MPP	Yes	PFC Sigma RP (rotating platform) (PS), PFC Sigma RP-F (rotating platform) (PS)	Yes	NA
Choi	32	(1) Mobile bearing group is better than medial-pivot fixed bearing group in satisfaction (2) Patients with flexion contracture are less satisfied	New KSS (40 points)	2016	South Korea	Cohort	Minimum 5 years	101	12	89	67.1	27.5	OA	MPP	Yes	52 ACS (mobile bearing), 49 Advance (fixed bearing)	Yes	General
Clement	33	Patients with poor mental health were less satisfied	4 grades (very satisfied, satisfied, neutral, unsatisfied)	2013	UK	Cohort	1 year	962	418	544	70.5	NA	OA	NA	Yes and no	Kinemax, PFC sigma, Triathlon	NA	NA
Clement	34	Patients with back pain were less satisfied	4 grades (very satisfied, satisfied, neutral, dissatisfied)	2013	UK	Cohort	1 year	2392	1017	1375	70.4	NA	OA	NA	NA	Kinemax, Triathlon, PFC Sigma	NA	NA
Clement	35	Diabetes melitus had no effect on satisfaction	4 grades (very satisfied, satisfied, uncertain, unsatisfied)	2013	UK	Cohort	1 year	2392	1014	1375	70.3	NA	OA	NA	NA	Kinemax, Triathlon, PFC Sigma	NA	NA
Clement	36	Patients with a subclinical improvement in their general physical well-being were less likely to be satisfied	4 grades (very satisfied, satisfied, neutral, unsatisfied)	2013	UK	Cohort	12 months	2330	996	1334	70.2	NA	OA	NA	NA	Kinemax, Triathlon, PFC Sigma	Yes	NA
Clement	37	Post-operative OKS positively related to satisfaction	4 grades (very satisfied, satisfied, neutral, unsatisfied)	2013	UK	Cohort	1 year	2392	1017	1357	70.4	NA	OA	NA	NA	Kinemax, PFC sigma, Triathlon	NA	NA
Clement	38	Pre-operative OKS and improvement in OKS positively related to satisfaction	4 grades (very satisfied, satisfied, unsure, unsatisfied)	2013	UK	Cross-sectional	1 year	966	421	545	70.6	NA	OA	NA	NA	Kinemax, PFC sigma, Triathlon	NA	NA
Clement	39	Using ASM navigation did not relate to satisfaction	4 grades (very satisfied, satisfied, uncertain, unsatisfied)	2017	UK	Cohort	1 year	295	121	174	68.4	31.0	OA	MPP	NA	NA	Yes	NA
Clement	40	Age and gender did not relate to satisfaction. The risk of dissatisfaction was significantly increased if a patient’s expectation was not achieved	4 grades (very satisfied, satisfied, neutral, unsatisfied)	2014	UK	Cohort	1 year	322	128	194	70.5	NA	OA	NA	NA	Kinemax, Triathlon, PFC Sigma	Yes	NA
Clement	41	No difference in gap balanced technique and measured resection technique in computer-navigated TKR	5 grades (very satisfied, satisfied, neutral, unsatisfied, very unsatisfied)	2017	UK	Cohort	5.4 years	144	65	79	69.0	31.2	NA	MPP	NA	Columbus	Yes	NA
Collados-Maestre	42	(1) Patients with pre-operative low back pain were less satisfied (2) Patients with severe low back pain were less satisfied than patients with moderate low back pain	VAS (0–10)	2016	Spain	Cohort	3.2 years	48	19	29	73.7	30.4	OA	MPP	Yes	Trekking (CR)	Hybrid (cemented tibia)	Spinal
Collados-Maestre	43	Single radius prosthesis group was better than multi radius prosthesis group	5 grades (very satisfied, satisfied, neutral, dissatisfied, very dissatisfied)	2016	Spain	Randomised controlled trial	5.7 years	237	72	165	71.0	31.0	OA	MPP	Yes	Trekking (fixed bearing) (CR, single-radius), Multigen (fixed bearing) (CR, multi-radius)	Hybrid (cemented tibia)	Spinal
Conditt	44	No difference between PS and CR	Total Knee Function Questionnaire	2004	USA	Cohort	1 year	49	21	28	70.5	NA	NA	NA	NA	AMK (21 PS, 28 CR)	NA	NA
Devers	45	Post-operative passive knee flexion did not relate to satisfaction	5 grades	2011	USA	Cross-sectional	4 years	122	29	93	69.0	30.8	OA, RA, post-trauma OA	NA	NA	PFC Sigma (PS)	NA	NA
Dixon	46	Patients with Triathlon were more satisfied than those with Kinemax Plus	4 grades	2014	UK	Cohort	12 months	453	150	303	69.0	NA	OA, RA	NA	Yes and no	Triathlon (fixed bearing) (92% CR, 8% PS), Kinemax plus (53% fixed bearing)	Yes	NA
Dhurve	47	(1) Age and BMI did not relate to satisfaction (2) Poor improvement of range of motion (ROM), pain catastrophizing and depression, severe swelling and unwilling to do post-operative rehabilitation programs related to dissatisfaction	5 grades (very satisfied, satisfied, neutral, dissatisfied or very dissatisfied)	2016	Australia	Cross-sectional	Minimum 1 year	301	142	159	73.9	30	NA	NA	NA	NA	NA	NA
Dickstein	48	Severe pain and inability to use the stairs related to dissatisfaction	Satisfied or not	1997	Israel	Cross-sectional	12 months	79	26	53	70	NA	OA	NA	NA	NA	Yes	NA
Duivenvoorden	49	Patients with pre-operative depressive or anxiety symptoms were less satisfied	5 grades	2013	Netherlands	Cohort	12 months	128	56	72	66.2	NA	OA	NA	NA	NA	NA	NA
Filardo	50	Control Preference Scale related to satisfaction	NRS (0–10)	2016	Italy	Cohort	12 months	176	56	120	66	28.0	OA	MPP	NA	NA	NA	NA
Franklin	51	Patients who used narcotics before TKA were more likely to be dissatisfied	Unclear	2010	USA	Cohort	12 months	6346	2065	4224	67.4	31.9	OA	NA	NA	NA	NA	NA
Fricka	52	No difference between cemented TKR and cementless TKR	Satisfied or not	2015	USA	Randomised controlled trial	2 years	99	37	62	59.3	32.0	NA	Subvastus	Yes	NexGen CR-flex (fixed bearing) (CR)	50 Yes 49 No	NA
Furu	53	Patients with greater knee extensor strength were more satisfied	New KSS (40 points)	2016	Japan	Cohort	1 year	30	4	24	73.6	25.5	OA, RA	MPP	Yes	Bi-surface, NexGen LPS-flex (fixed bearing) (PS)	Yes	NA
Giurea	54	Patients with specific personality traits (life satisfaction, performance orientation and emotional stability) were more satisfied	Satisfied or not	2016	Austria	Cohort	Minimum 2 years	70	32	48	66.0	NA	OA	MPP	Yes	E.motion UC (rotating platform) (CR)	Yes	NA
Gong	55	Significantly different satisfaction rate amongst the four personality: choleric type, 74.2%; sanguine type, 92.3%; melancholic type, 81.2%; phlegmatic type, 87.3%	VAS (0–100)	2014	China	Cross-sectional	6 months	387	109	278	59.6	27.8	OA	NA	NA	Gemini MK II	NA	Epidural or nerve block
Goodman	56	No difference between RA patients and OA patients	5 grades	2016	USA	Cohort	2 years	4456	1852	2604	67.1	30.7	OA, RA	NA	NA	NA	NA	NA
Goudie	57	Patients with post-operative flexion contracture of 5 degrees or greater were less satisfied	4 grades (very satisfied, satisfied, unsure, dissatisfied)	2011	UK	Cohort	2 years	811	317	489	69.0	30.5	OA	NA	NA	Unknown (779 CR, 32 PS)	NA	NA
Gustke	58	By using Orthosensor, 96.7% in the medial-lateral balanced group and 82.0% in the unbalanced group were satisfied	5 grades	2014	USA	Cohort	1 year	137	47	90	71.0	30.5	OA	MPP, subvastus, midvastus	Yes	NA	Yes	NA
Ha	59	Patients with greater improve in ROM following TKR were more satisfied	4 grades (very satisfied, somewhat satisfied, somewhat dissatisfied, very dissatisfied)	2016	South Korea	Cohort	3.2 years	630	58	572	66.2	26.7	OA, RA, AVN	NA	No	206 NexGen LPS-flex (PS), 163 Genesis II, 160 Triathlon, 101 Vanguard	NA	NA
Hamilton	60	Patients using Triathlon prosthesis were more satisfied than those using Kinemax prosthesis	4 grades (very satisfied; satisfied; unsure, dissatisfied)	2015	UK	Randomised controlled trial	3 years	212	81	131	69.0	NA	OA	NA	No	Triathlon (fixed bearing) (CR), Kinemax (fixed bearing) (CR)	Yes	NA
Harvie	61	Computer-navigated TKA did not relate to satisfaction	5 grades	2010	Australia	Randomised controlled trial	5 years	46	18	28	70.1	NA	OA and RA	NA	No	NA	NA	NA
Hawker	62	Less education and greater BMI negatively related to satisfaction	5 grades	1998	Canada, USA	Cross-sectional	Minimum 2 years	1193	344	849	72.6	NA	OA, RA, post-trauma OA, other	NA	NA	NA	NA	NA
Heesterbeek	63	No difference between fixed and mobile bearing	NRS (0–10)	2016	Netherlands	Cross-sectional	10 years	189	52	106	67.1	28.6	OA	NA	Yes and no	NA	NA	NA
Hernandez-Vaquero	64	Minimally invasive surgery had no effect on satisfaction	VAS (0–10)	2010	Spain	Randomised controlled study	6 months	62	11	51	70.6	31.5	OA	Mini-midvastus, MPP	Yes	Triathlon (CR)	Yes	NA
Hinarejos	65	No difference between single radius prosthesis and multi-radius prosthesis	VAS (0–10)	2016	Spain	Cohort	5 years	474	126	348	72.2	31.3	OA	MPP	Yes	Triathlon (PS, single-radius), Genutech (PS, multi-radius)	Yes	NA
Hirschmann	66	Lateral subvastus approach related to better satisfaction	VAS (0–10)	2010	Switzerland	Cohort	2 years	143	55	88	69	30	OA	Lateral parapatellar approach, subvastus approach, or MPP	Yes and No	NA	Yes or hybrid	NA
Hui	67	No difference between oxidised zirconium and cobalt–chromium femoral components	British Orthopaedic Association grading system	2011	Australia	Randomised controlled trial	5 years	80	15	25	NA	NA	OA	MPP	Yes	Genesis II	Yes	Spinal and/or epidural
Huijbregts	68	(1) Coronal alignment of the femoral component was 0.5 degrees more accurate (*P* < 0.05) in patients who were satisfied (2) Dissatisfaction was associated with OKS	5 grades (very satisfied, satisfied, neutral/not sure, dissatisfied, very dissatisfied)	2016	Australia	Cohort	1 year	230	105	106	69.0	30.2	OA, RA, AVN, unknown	MPP, lateral parapatellar	Yes and no (including patellectomy)	Genesis II, Legion, ACS (139 CR, 91 PS)	NA	NA
Hwang	69	Patellar resurfacing did not relate to satisfaction	Satisfied or not	2011	South Korea	Case–control	7 years	275	6	264	68	26.5	OA	MPP	Yes and no	LCS (mobile bearing)	Yes	NA
Jacobs	70	Patients with intact ACL (at the time of CR TKR) were less satisfied	3 grades (satisfied, I'm not sure, dissatisfied)	2016	USA	Cohort	5.1 years	562	183	379	65.0	34.0	NA	NA	NA	Vanguard Mono-lock (CR)	NA	NA
Jacobs	71	(1) African American patients were 3.0 times more likely to be dissatisfied than Caucasians (2) Patients with mild degenerative changes were 2.1 times more likely to be dissatisfied than patients with severe degenerative changes	3 grades (yes (satisfied), I'm not sure, no)	2014	USA	Cross-sectional	3.5 years	989	326	663	65.0	34.3	OA	MPP	Yes	Unknown (CR)	NA	NA
Jacobs	72	(1) No difference in age, gender and BMI between satisfied patients and dissatisfied patients (2) Satisfied patients showed greater improvement in ROM, Knee Society pain score and Knee Society function score than dissatisfied patients	4 grades (yes (satisfied), I'm not sure, no)	2014	USA	Cross-sectional	2.8 years	768	247	521	65.0	34.3	OA	NA	Yes	Vanguard complete femoral component with Monolock tibial component (CR)	NA	NA
Jacobs	73	Patients with movement-elicited pain or pain at rest were less satisfied	3 grades (yes (satisfied), I'm not sure, no)	2015	USA	Cohort	3.8 years	316	91	184	65.1	33.9	OA	NA	NA	Unknown (CR)	NA	NA
Jacobs	74	Patients with intra-operative greater forces (> 10 lbf) in the medial compartment than in the lateral compartment in extension were more satisfied	Satisfied or not	2016	USA	Cohort	6 months	50	21	29	66.1	34.5	OA	MPP	NA	Vanguard (CR), Persona (CR)	NA	NA
Jain	75	Patient satisfaction was higher in the Vega and Genesis II groups than the E.motion group	British Orthopaedic Association grading system	2017	UK, South Korea, India	Cohort	2 years	627	30	597	69.6	27.3	OA	MPP	Yes	Vega-PS, E.motion-PS, Genesis II	Yes	NA
Kaneko	76	The varus ligament balance with 30, 60 degrees of flexion negatively correlated with satisfaction	New KSS (40 points)	2016	Japan	Case series	2 years	39	8	31	78	24.4	OA	NA	NA	Bi-cruciate stabilised substituting (BCS) prosthesis	Yes	NA
Kawahara	77	(1) Patients with internal rotation of the femoral component greater than 3 degrees relative to the surgical epicondylar axis were less satisfied (2) Internal or external malrotation of tibial component had no effect on satisfaction	New KSS (40 points)	2014	Japan	Cross-sectional	3.9 years	92	NA	NA	75.7	25.6	OA	NA	Yes	NexGen LPS-flex (fixed bearing) (PS)	NA	NA
Kawakami	78	No significant difference between CR and PS	New KSS (40 points)	2015	Japan	Randomised controlled trial	98 months	48	8	40	74.2	NA	OA	MPP	NA	NexGen CR-flex (CR), NexGen LPS-flex (PS)	Na	NA
Keurentjes	79	Patients with severe radiographic OA (K/L grades 3, 4) were more satisfied than patients with mild radiographic OA (K/L grades 0, 1 and 2)	NRS (0–10)	2013	Netherlands	Cohort	2.82 years	278	86	192	69.2	NA	OA	NA	NA	NA	NA	NA
Keurentjes	80	Completed level of schooling had no effect on satisfaction	NRS (0–10)	2013	Netherlands	Cohort	3.16 years	262	88	174	67.7	NA	OA	NA	NA	NA	NA	NA
Khamis	81	No difference between Scorpio NRG CR and PFC Sigma CR	Satisfied or not	2013	Bahrain	Cohort	1 year	299	145	154	65.9	NA	OA	MPP	NA	Scorpio NRG (CR), PFC Sigma (CR)	NA	NA
Kim	82	Patients with medial pivot fixed bearing prosthesis were less satisfied than those with PFC Sigma mobile bearing prosthesis	VAS (0–10)	2008	South Korea	Randomised controlled study	2.6 years	184	7	85	69.5	27.8	OA	MPP	Yes	Advance (fixed bearing) (CR), PFC Sigma (mobile bearing) (CR)	Yes	NA
Kim	83	Patients with rotating platform (E.motion RP) were more satisfied than those with floating platform (E.motion FP)	4 grades (enthusiastic, satisfied, not committed, disappointed)	2009	South Korea	Cohort	24 months	186	9	177	68.5	26.3	NA	MPP	Yes	93 E.motion FP (CR), 93 E.motion RP (PS)	Yes	NA
Kim	84	No difference between gender-specific LPS-flex and conventional LPS-flex	VAS (0–10)	2010	South Korea	Randomised controlled study	2.13 years	170	0	85	69.7	27.1	OA	MPP	Yes	LPS-flex (gender specific, conventional) (PS)	Yes	NA
Kim	85	No difference between patients with patellar resurfacing and those without it using high-flexion prosthesis	5 grades (fully satisfied, satisfied, barely satisfied, dissatisfied, very dissatisfied)	2014	South Korea	Cohort	Minimum 7 years	92	8	84	66.2	27.0	OA	MPP	Yes and No	NexGen LPS-flex (fixed bearing) (PS)	Yes	NA
Kim	86	Poor pre-operative WOMAC pain score and post-operative decrease in range of motion negatively related to dissatisfaction	4 grades (enthusiastic, satisfied, noncommittal, disappointed)	2009	South Korea	Cross-sectional	Minimum 12 months	438	9	261	68.4	26.4	OA	MPP	Yes	Genesis II (fixed bearing), E.motion (mobile bearing)	Yes	NA
Kim	87	No difference between NexGen CR-flex and NexGen CR	VAS (0–10)	2009	South Korea	Randomised controlled study	3.13 years	108	5	49	69.7	26.7	OA	MPP	Yes	NexGen (CR), NexGen CR-flex (CR)	Yes	NA
Kim	88	No difference between standard NexGen CR-flex and gender-specific NexGen CR-flex	VAS (0–10)	2010	South Korea	Randomised controlled study	3.25 years	276	0	138	71.2	27.3	OA	NA	Yes	NexGen CR-flex (gender specific, conventional) (CR)	Yes	NA
Kim	89	Dissatisfied patients tended to perceive high flexion activities to be more important than satisfied patients	4 grades (enthusiastic, satisfied, not committed, disappointed)	2010	South Korea	Cross-sectional	Minimum 12 months	261	0	261	68.4	26.7	OA	MPP	Yes	216 Genesis II (fixed bearing), 208 E.motion (mobile bearing)	Yes	NA
Kim	90	No significant influence by post-operative leg length discrepancy	5 grades (fully satisfied, satisfied, barely satisfied, dissatisfied, very dissatisfied)	2015	South Korea	Cohort	30 months	148	15	133	69.5	26.6	OA	Midvastus	No	Columbus (PS)	Yes	NA
Kim	91	PFC CR mobile-bearing Sigma were better than Medial-Pivot knee prosthesis about satisfaction	4 grades	2017	South Korea	Randomised controlled study	12.1 years	364	52	130	65.6	29.8	OA	MPP	Yes	Medial-Pivot (PS), PFC Sigma CR	Yes	NA
Kim	92	Cement use did not relate to satisfaction	VAS (0–10)	2013	South Korea	Randomised controlled study	16.6 years	160	17	63	54.3	27.8	OA	MPP	Yes	NexGen CR	Yes	NA
Kim	93	Using a highly cross-linked polyethylene did not relate to satisfaction in PS TKR	VAS (0–10)	2014	South Korea	Case–control	5.9 years	308	20	288	60.3	29.1	OA	MPP	Yes	Yes	Yes	NA
Klit	94	There were no statistically significant differences in the outcome of pre-operatively depressed and non-depressed patients concerning satisfaction	5 grades (very satisfied, satisfied, neutral, dissatisfied and very dissatisfied)	2013	Denmark	Cohort	12 months	115	54	61	54	NA	OA	MPP	NA	CR, fixed (AGC, PFC, Triathlon), CR, rotating bearing (PFC-Sigma Vanguard ROCC, NexGen), PS, fixed (LPS-flex)	NA	NA
Kornilov	95	The patients who reported ‘very good’ overall satisfaction tended to be younger	5 grades	2017	Russia, Norway	Cohort	1 year	79	4	65	63	NA	OA	MPP	NA	NA	Yes	Spinal
Kosse	96	Satisfaction did not improve by using patient-specific instrumentation	VAS (0–10)	2017	Netherlands	Randomised controlled trial	12 months	42	20	22	63.1	27.95	OA	MPP	Yes	Genesis II (PS, fixed)	Yes	
Kotela	97	No difference between patient-specific CT-based instrumentation (signature) and conventional	VAS (0–100)	2015	Poland	Randomised controlled trial	12 months	95	29	66	66.3	29.8	OA	MPP	No	Vanguard (CR)	No	NA
Krushell	98	85% of patients with BMI > 40 were satisfied and 95% of patients with BMI < 30 were satisfied	Satisfied or not	2007	USA	Case–control	90 months	78	NA	NA	68.1	35.0	OA	MPP, midvastus	Yes	Osteonics series 3000, Osteonics series 7000, Scorpio	Yes	NA
Khuangsirikul	99	Computer-assisted TKA did not relate to satisfaction	Original questionnaire	2016	Thailand	Cohort	10 years	144	14	130	76.9	NA	OA	NA	NA	NA	NA	NA
Kuriyama	100	Post-operative noise had no relation to satisfaction	New KSS (40 points)	2016	Japan	Cross-sectional	12 months	35	NA	NA	NA	NA	OA, RA. AVN	NA	NA	Bi-surface (fixed bearing) (PS)	NA	NA
Kuroda	101	No item in pre-operative new Knee Society Scores (objective knee indicators, symptoms, satisfaction, expectations, functional activities) had impact on satisfaction	New KSS (40 points)	2016	Japan	Cohort	1 year	79	12	63	74.8	NA	OA, AVN, RA	NA	NA	PFC Sigma, e-motion	NA	NA
Kwon	102	Generalised joint laxity did not relate to satisfaction	VAS (0–10)	2016	South Korea	Case–control	3 years	338	0	338	68	25.9	OA	MPP	Yes	PFC	Yes	NA
Kwon	103	Intra-operative periarticular injection with corticosteroid did not improve satisfaction	VAS (0–10)	2013	South Korea	Randomised controlled trial	6 months	76	0	76	69.3	25.9	OA	MPP	No	PFC sigma PS	Yes	NA
Lehnen	104	Computer-assisted TKR was better than conventional TKR regarding satisfaction	5 grades (extremely satisfied, very satisfied, moderately satisfied, slightly satisfied, not at all satisfied)	2011	Switzerland	Cohort	12 months	165	59	106	70.0	NA	NA	MPP	NA	LCS (mobile bearing)	Yes	NA
Li	105	Continuous irrigation of 4000 ml cold saline with 0.5% epinephrine group was better than normal temperature solution group	VAS (0–10)	2016	China	Cohort	60 h	389	53	336	61.0	28.7	OA	NA	NA	Gemini Link (CR)	Yes	Epidural or nerve block
Lim	106	No difference between patients with and without history of previous knee surgery (anterior cruciate ligament reconstruction or high tibial osteotomy)	6 grades (excellent, very good, good, fair, poor; terrible)	2016	Singapore	Cross-sectional	2 years	303	220	83	65.0	27.2	OA	MPP	NA	NA	NA	NA
Lingard	107	No difference amongst TKRs undertaken in the USA, UK and Australia	4 grades (very satisfied to very dissatisfied)	2006	USA, UK, Australia	Cohort	12 months	598	254	344	69.3	29.3	OA	NA	Yes and no	Kinemax	Yes	NA
Liow	108	No difference between iASSIST computer-assisted stereotaxic navigation group and conventional group	6 grades	2016	Singapore	Case–control	6 months	192	53	139	65.5	27.9	OA	MPP	NA	NA	NA	NA
Liow	109	No difference between robotic-assisted TKR and conventional TKR	6 grades	2016	Singapore	Randomised controlled trial	2 years	60	NA	NA	67.9	NA	OA	MPP	Yes	NexGen LPS-flex (PS)	NA	NA
Lizaur-Utrilla	110	Patients with mobile bearing insert were more satisfied than those with fixed bearing insert	VAS (0–10)	2012	Spain	Randomised controlled trial	2 years	119	25	94	74.2	32.0	OA	MPP	Yes	Trekking mobile bearing (CR), Multigen Plus fixed bearing (CR)	Hybrid (cemented tibia)	Epidural
Lizaur-Utrilla	111	Dissatisfaction rate was higher in patients waiting longer than 6 months	5 grades (very satisfied, satisfied, neutral, dissatisfied, very dissatisfied)	2016	Spain	Cohort	1 year	192	65	127	69.7	30.7	OA	MPP	Yes	Trekking	Hybrid (cemented tibia)	Spinal
Lizaur-Utrilla	112	Satisfaction was higher in the octogenarian group than the septuagenarian	VAS (0–10)	2016	Spain	Cohort	3.2 years	292	212	80	83.1 G1 75.2 G2	30.2	OA	NA	Yes	Yes	Hybrid	Epidural
Losina	113	Patients having a lack of hospital choice were less satisfied	4 grades (very satisfied, somewhat satisfied, somewhat dissatisfied, very dissatisfied)	2005	USA	Cross-sectional	2 years	932	308	624	74.0	NA	OA, other	NA	NA	NA	NA	NA
Lygre	114	(1) Patella resurfacing did not relate to satisfaction (B) Patients with NexGen were more satisfied than those with AGC	VAS (0–100)	2010	Norway	Case–control	7.1 years	972	281	691	76.0	NA	OA	NA	Yes and No	AGC (CR), Genesis I (CR), NexGen (CR), LCS (CR)	NA	NA
Machhindra	115	No difference between Ultra Congruent prosthesis and PS prosthesis	4 grades (enthusiastic, satisfied, noncommittal, disappointed)	2015	South Korea	Cohort	2 years	281	10	219	80.0	27.4	OA	MPP	Yes	E.motion ultra-congruent (mobile bearing) (UC), E.motion (mobile bearing) (PS)	Yes	NA
Maddali	116	No difference between outcomes of one-stage and two-stage TKR for bilateral knee arthritis	4 grades (very satisfied, satisfied, unsure, dissatisfied)	2015	China	Cohort	2.4 years	278	46	93	68.9	24.0	OA, RA	MPP	No	Gemini MK II (mobile bearing) (PS)	Yes	General
Mannion	117	Patients with problems in other joints and poor improvement in symptoms and function were less satisfied	4 grades (very satisfied, somewhat satisfied, somewhat dissatisfied, very dissatisfied)	2009	Switzerland	Cross-sectional	2 years	112	34	78	67.0	NA	OA	NA	NA	NA	NA	NA
Matsuda	118	Old age and varus post-operative alignment negatively related to satisfaction	New KSS (40 points)	2013	Japan	Cross-sectional	5 years	375	64	311	71.0	26.0	OA, RA, other	NA	Yes	Unknown (82% PS, 18% CR)	NA	NA
Matsumoto	119	Patient satisfaction exhibited positive correlations with joint component gap difference	New KSS (40 points)	2017	Japan	Cohort	1 year	35	6	29	75.5	NA	OA	MPP	NA	E-motion floating platform mobile-bearing CR	NA	NA
Mayman	120	More patients were extremely satisfied with patellar resurfacing	4 grades (extremely satisfied, satisfied, unsure, or disappointed)	2003	Canada	Randomised controlled trial	2 years	100	42	58	72	NA	OA	NA	Yes and no	NA	Yes	NA
McLawhorn	121	Patients with reported allergies were less satisfied	3 grades (somewhat to very satisfied, neither satisfied or dissatisfied, somewhat to very dissatisfied)	2015	USA	Cohort	2 years	257	119	138	67.5	30.1	NA	NA	NA	Unknown (PS)	Yes	NA
Meftah	122	No significant difference between rotating platform and fixed bearing	VAS (0–10)	2016	USA	Cohort	12.3 years	55	16	24	54.3	31.8	OA, RA, post-trauma OA	MPP	Yes	PFC Sigma (20 rotating platform, 34 fixed bearing) (PS)	Yes	NA
Meijerink	123	Patients with PFC prosthesis were more satisfied than those with CKS prosthesis	VAS (0–100)	2011	Netherlands	Randomised controlled trial	5.6 years	77	27	50	67.0	29.0	OA, RA	MPP	No	PFC (fixed bearing) (CR), CKS (fixed bearing) (CR)	Yes	NA
Meijerink	124	There was no relation between surgeon’s pre-operative assessment of the difficulty or surgeon’s immediate post-operative satisfaction and patient’s satisfaction	VAS (0–100)	2009	Netherlands	Cohort	1 year	53	15	36	67.0	NA	OA, RA	NA	NA	PFC, CKS	NA	NA
Merle-Vincent	125	Absence of complications, BMI less than 27, high radiological joint narrowing score, age greater or equal to 70 years and absence of depression positively related to satisfaction	5 grades (0, 25, 50, 75, 100% of satisfaction)	2011	France	Cohort	2 years	264	78	186	75.0	28.4	OA	NA	NA	NA	NA	NA
Miner	126	(1) WOMAC pain score and WOMAC function score were positively related to satisfaction (2) Knee flexion angle, age, gender and BMI did not relate to satisfaction	4 grades	2003	UK	Cohort	12 months	684	283	401	69.8	29.5	OA	NA	NA	Kinemax	NA	NA
Mistry	127	Presence of altered sensation did not affect satisfaction	British Orthopaedic Association grading system & VAS (0–10)	2005	New Zealand	Cohort	1 year	29	8	21	72.7	NA	NA	NA	NA	NA	NA	NA
Mont	128	Patient’s pre-operative activity level did not relate to satisfaction	VAS (0–10)	2007	USA	Cohort	7 years	144	44	70	70.0	29.0	OA, RA, AVN	NA	NA	Duracon (CR)	NA	NA
Murphy	129	No difference between patients with femoral component implanted in 4 degrees flexion in the sagittal plane and those with femoral component implanted in a neutral position	NRS (0–10)	2014	Australia	Randomised controlled trial	1 year	40	15	25	70.3	30.5	OA	MPP	No	Profix (CR)	NA	NA
Nakahara	130	Post-operative ability of climbing up or down a flight of stairs, getting into or out of a car, moving laterally (stepping to the side) and walking and standing effected on satisfaction	New KSS (questions 3, 4, 5 only)	2015	Japan	Cross-sectional	5 years	520	62	325	72.0	NA	OA, RA, AVN	NA	NA	Unknown (82% PS, 18% CR)	NA	NA
Nakano	131	Use of CT-free navigation had no effect on satisfaction	New KSS (40 points)	2013	Japan	Cohort	118 months	27	3	24	71.5	NA	OA	MPP	NA	PFC Sigma (PS)	NA	NA
Nam	132	Patients with metallic allergy were less satisfied	New KSS (40 points)	2016	USA	Cohort	Minimum 2 years	589	226	363	62.3	32.9	NA	NA	NA	NA	NA	NA
Nam	133	(1) Female patients, patients from low-income households (< USD 25,000 annually) were less satisfied (2) Education level, employment status and using custom cutting guides, gender-specific prosthesis, high-flex prosthesis, rotating platform bearing or kinematic alignment technique had no effect on satisfaction	Satisfied or not	2014	USA	Cross-sectional	2.6 years	661	NA	NA	54.3	NA	OA	NA	NA	Vanguard	Yes	NA
Nam	134	Using custom cutting guides (signature) had no effect on satisfaction	Satisfied or not	2016	USA	Cohort	3 years	448	154	294	61.9	NA	OA	Midvastus	Yes	Vanguard (fixed bearing) (CR)	Yes	NA
Narayan	135	Deep knee flexion did not relate to patient satisfaction after TKR (even in a population where squatting and sitting cross-legged are part of the normal lifestyle)	5 grades (extremely satisfied, satisfied, neutral, unsatisfied, extremely unsatisfied)	2009	India	Cohort	25.12 months	36	10	17	58.7	NA	OA	NA	NA	PFC, Genesis II (23 CR, 13 PS)	NA	NA
Nishio	136	Regarding intra-operative kinematic patterns, medial pivot group were more satisfied than non-medial pivot group	New KSS (40 points)	2014	Japan	Cross-sectional	42 months	40	8	32	73.0	25.6	OA	Subvastus	Yes	PFC Sigma RP-F (mobile bearing) (PS)	NA	NA
Noble	137	Age less than 60, absence of residual symptoms, fulfilment of expectations and absence of functional impairment positively related to satisfaction	Total Knee Function Questionnaire	2006	USA	Cross-sectional	Minimum 1 year	253	105	148	68.1	NA	OA, RA, post-trauma OA	NA	NA	NA	NA	NA
Nunez	138	Post-operative WOMAC score related to satisfaction	5 grades	2009	Spain	Cohort	7 years	112	26	86	67.3	30.7	OA	NA	NA	NA	NA	NA
Nunley	139	In CR TKR, rotating platform, gender-specific design and high flex design had no effect on satisfaction (compared with conventional CR prosthesis)	Satisfied or not	2015	USA	Cohort	2.6 years	527	196	331	55.6	NA	OA, post-trauma OA, AVN	NA	NA	Vanguard (CR), unknown (rotating platform (CR, PS), gender-specific (CR), high-flex (CR))	NA	NA
Park	140	In simultaneous bilateral TKR, there was no difference between cemented and cementless TKR	VAS (0–10)	2011	South Korea	Randomised controlled trial	13.6 years	100	11	39	58.4	26.6	OA, inflammatory disease	MPP	Yes	NexGen (CR)	Yes and no	NA
Parsley	141	No difference between PS and ultra-congruent prosthesis	Total Knee Function Questionnaire	2006	USA	Cohort	Minimum 2 years	209	61	148	67.9	29.9	NA	Midvastus	NA	Sulzer Apollo (PS), Sulzer NK-II Ultra-congruent	Yes	NA
Perez-Prieto	142	Pre-operative depression had no effect on satisfaction	Satisfied or not	2014	Spain	Cohort	1 year	716	550	166	72.5	31.4	NA	NA	NA	NA	NA	NA
Pulavarti	143	Patients with patella denervation were more satisfied	4 grades (excellent, good, fair, poor)	2014	UK	Randomised controlled trial	26.4 months	126	58	68	69.9	29.2	OA	MPP	No	Unknown (CR)	NA	NA
Ranawat	144	No difference between fixed bearing and rotating platform	VAS (0–10)	2004	Italy	Cohort	46 months	52	9	17	74.0	NA	OA, RA	NA	Yes	PFC Sigma (mobile bearing and fixed bearing) (PS)	Yes	NA
Ranawat	145	No difference between Attune PS and PFC Sigma PS	VAS (0–10)	2016	USA	Cohort	2 years	200	62	138	70.6	29.3	OA	MPP	Yes	100 Attune (61 fixed bearing, 39 rotating platform) (PS), 100 PFC Sigma (83 fixed bearing, 17 rotating platform) (PS)	Yes	NA
Razmjou	146	Patients with neuropathic pain were less satisfied	6 grades (very satisfied, somewhat satisfied, a little bit satisfied, a little bit dissatisfied, somewhat dissatisfied, very dissatisfied)	2015	Netherlands	Cross-sectional	5 years	63	16	47	67.0	NA	OA	NA	NA	NA	NA	NA
Roberts	147	(1) Male patients and patients with OA were less satisfied (2) Age had no effect on satisfaction	Satisfied or not	2007	UK	Cross-sectional	15 years	912	NA	NA	69.5	NA	OA, RA, other	NA	Yes and No	Freeman-Samuelson, Insall Burstein II, Kinematic, Kinemax, Omnifit, PFC	NA	NA
Roberts	148	Patients with patellar resurfacing were more satisfied than those without it	5 grades	2015	USA	Randomised controlled trial	10 years	327	170	157	70.6	29.2	OA	MPP	Yes and No	PFC Sigma (fixed bearing) (CR)	NA	Spinal
Robertsson	149	(1) Women gender, not chronic pain, old age and non-patellar resurfacing negatively related to satisfaction (2) Satisfaction rate was RA > OA > post-trauma arthritis > AVN	4 grades (very satisfied, satisfied, uncertain, dissatisfied)	2000	Sweden	Cross-sectional	6 years	27372	NA	NA	71.0	NA	OA RA, ON, other	NA	Yes and no	NA	NA	NA
Schlegel	150	Patients with surface-cemented tibial component were more satisfied than patients with fully cemented tibial component	5 grades	2015	Germany	Cohort	11.4 years	67	4	63	66.0	NA	RA, OA	MPP	Yes	PFC (fixed bearing) (CR)	Yes (25 surface only, 42 fully cemented)	NA
Schnurr	151	Patients with mild to moderate OA were less satisfied	5 grades (completely satisfied, partially satisfied, neutral, partially unsatisfied, completely unsatisfied)	2013	Germany	Cohort	2.8 years	996	338	658	68.0	NA	OA	MPP	NA	PFC Sigma, NexGen high-flex	NA	NA
Schuster	152	Post-operative anterior–posterior stability had no effect on satisfaction	VAS (0–10)	2011	Switzerland	Cohort	47.2 months	127	32	80	70.7	29.3	NA	NA	NA	balanSys (fixed bearing) (CR)	NA	NA
Scott	153	Poor OKS, poor pre-operative SF-12 mental component score, depression, back pain and pain in other joints negatively related to satisfaction	4 grades (very satisfied, satisfied, unsure, dissatisfied)	2010	UK	Cohort	12 months	1141	515	698	70.1	NA	OA	NA	No	PFC Sigma (CR), Kinemax (CR), Triathlon (CR)	NA	NA
Scott	154	In staged bilateral TKR, satisfaction on the first side was not always translated into that of the other side	4 grades (very satisfied, satisfied, uncertain, dissatisfied)	2014	UK	Cohort	12 months	70	30	40	71.7	NA	OA, inflammatory disease	NA	NA	NA	NA	NA
Scott	155	No difference between TKR for primary OA and post-trauma (tibial plateau fracture) OA	4 grades (very satisfied, satisfied, uncertain, dissatisfied)	2015	UK	Cohort	Minimum 5 years	124	32	92	66.0	NA	OA, post-trauma OA (tibial plateau fracture)	MPP	NA	Unknown (CR)	Yes	NA
Scott	156	Poor pre-operative OKS, poor improvement in OKS and post-operative stiffness (in patients under 55 years) independently predicted dissatisfaction	4 grades (very satisfied, satisfied, unsure, dissatisfied)	2016	UK	Cohort	12 months	177	78	99	50.0	34.0	OA, post-trauma OA, inflammatory disease	NA	No	109 Triathlon (CR), 63 PFC Sigma (CR), 4 Kinemax (CR), 1 hinged implant	NA	NA
Senioris	157	Patellar congruence had no effect in mobile-bearing TKR	4 grades (excellent, good, fair, poor)	2016	France	Cohort	14 months	30	8	22	68.8	31.2	OA	Midvastus	No	HLS KneeTec (mobile bearing) (PS)	No	General
Seo	158	Octogenarians had same level of satisfaction as young patients	NRS (0–10)	2015	South Korea	Cohort	1 year	757	68	689	81.9 G1 67.7 G2	28.8	OA	MPP	Yes	NA	Yes	NA
Sharkey	159	Combination of post-operative noise and numbness negatively related to satisfaction	5 grades (completely satisfied, partially satisfied, neutral, partially unsatisfied, completely unsatisfied)	2011	USA	Cross-sectional	15 months	49	24	25	68.0	31.6	OA	NA	NA	NA	NA	NA
Shukla	160	No difference between MPP and midvastus approach	New KSS (40 points)	2016	India	Cohort	1 year	52	22	30	61.4	NA	NA	MPP, midvastus	NA	Genesis II (PS)	NA	NA
Singisetti	161	No difference between navigation (articular surface mounted (ASM) navigation technique) and conventional technique	4 grades (very satisfied, somewhat satisfied, somewhat dissatisfied, very dissatisfied)	2015	UK	Cohort	2 years	355	151	204	67.3	30.0	NA	NA	NA	Triathlon	NA	NA
Stickles	162	BMI did not relate to satisfaction	5 grades (very satisfied, somewhat satisfied, neutral, somewhat dissatisfied, very dissatisfied)	2001	USA	Cross-sectional	1 year	1011	374	637	69.9	31.2	OA	NA	NA	NA	NA	NA
Sun	163	Patelloplasty is better than traditional patellar management	Original questionnaire	2012	China	Cohort	55 months	152	72	80	64.7	NA	OA	MPP	No	PFC Sigma	Yes	NA
Thambiah	164	Post-operative WOMAC function scores, post-operative WOMAC final scores, improvements in the physical health component of the SF-36 score, and expectations being met were the factors which effect satisfaction	5 grades (extremely satisfied, satisfied, neutral, dissatisfied, extremely dissatisfied)	2015	Singapore	Cohort	1 year	110	32	78	64.0	26.7	OA	NA	NA	NA	NA	NA
Thomsen	165	No difference between standard CR prosthesis and high flexion PS prosthesis	VAS (0–10)	2013	Denmark	Randomised controlled trial	1 year	66	14	19	67.2	29.4	OA, RA	MPP	Yes	AGC (CR), NexGen LPS-flex (PS)	Yes	Combined (spinal + epidural)
Thomsen	166	No difference between gender-specific TKR and LPS-flex	VAS (0–10)	2011	Denmark	Randomised controlled trial	1 year	48	0	24	66	29.3	OA	MPP	NA	Gender Solutions high-flex prosthesis in one knee and a NexGen LPS-flex prosthesis in the other knee	Yes	Spinal
Tsukiyama	167	(1) Medial joint laxity made patients less satisfied (2) Lateral joint laxity did not affect satisfaction	New KSS (40 points)	2017	Japan	Cross-sectional	57 months	50	10	31	73	NA	OA	NA	NA	NA	NA	NA
van der Ven	168	No difference between high-flex prosthesis and conventional prosthesis	VAS (0–10)	2017	Netherlands	Randomised controlled trial	1 year	48	25	23	65	31.5	OA, RA	NA	NA	NA	NA	NA
van de Groes	169	Patients with femoral component medial malpositioned more than 5 mm were more satisfied	NRS (0–10)	2014	Netherlands	Cross-sectional	105.6 months	40	NA	NA	75.7	31.0	OA, RA	NA	No	LCS, PFC	NA	NA
van Houten	170	Patients with post-operative anterior knee pain were less satisfied	VAS (0–10)	2016	Netherlands	Cohort	10 years	60	15	45	63.7	NA	OA	NA	No	balanSys (43 fixed bearing, 17 AP-glide bearing) (CR)	NA	NA
Vissers	171	Pre-operative functional capacity and level of daily activity had no effect on satisfaction	5 grades (very satisfied, moderately satisfied, neutral, moderately dissatisfied, very dissatisfied)	2010	Netherlands	Cross-sectional	6 months	44	20	24	63.5	30.8	OA	NA	NA	Genesis II	NA	NA
Von Keudell	172	Amongst 3 age groups (54 or younger, 55 to 64, 65 or older), 65 or older group tended to be more satisfied than others	NRS (0–10)	2014	USA	Cohort	6.4 years	245	80	165	62.6	NA	OA	NA	NA	PFC Sigma	NA	NA
Wang	173	No difference between post-operative continuous femoral nerve block and patient-controlled epidural analgesia	4 grades (excellent, good, general, poor)	2015	China	Randomised controlled trial	12 months	162	NA	NA	NA	NA	NA	NA	NA	NA	NA	General
Waters	174	Patients with patellar resurfacing were more satisfied than those without it	4 grades	2003	UK	Randomised controlled trial	5.3 years	474	157	233	69.1	NA	OA, RA, inflammatory disease	MPP	Yes and no	PFC	NA	General
White	175	Amongst custom prosthesis (iTotal, cemented, CR), PFC Sigma (cemented, PS, fixed bearing) and PFC Sigma (non-cemented, CR, rotating platform), patients with custom prostheses were worst in satisfaction	VAS (0–10)	2016	USA	Cohort	2 years	74	31	43	52.2	NA	OA	MPP	NA	iTotal (CR), PFC Sigma (rotating platform) (CR), PFC Sigma (fixed bearing) (PS)	NA	NA
Williams	176	(1) Knee Society pain score, OKS, SF-12 (physical/mental), and knee flexion angle positively related to satisfaction (2) Age, BMI, length of stay, gender, diagnosis had no effect on satisfaction	4 grades (very happy, happy, OK (not perfect), never happy)	2013	UK	Cross-sectional	12 months	486	172	314	70.9	31.1	OA, RA	NA	NA	LCS (mobile bearing), ROCC (mobile bearing)	NA	NA
Wylde	177	No difference between fixed bearing and mobile bearing	4 grades	2008	UK	Randomised controlled trial	2 years	250	110	132	68.0	NA	OA, RA	NA	Yes and no	Kinemax plus (fixed bearing, mobile bearing)	NA	NA
Yagishita	178	Patients with high flexion PS prosthesis were more satisfied than those with high flexion CR prosthesis in simultaneous bilateral TKR	VAS (0–100)	2012	Japan	Randomised controlled trial	5 years	58	4	25	74.3	26.3	OA	NA	NA	NexGen CR-flex, NexGen LPS-flex	NA	NA
Yeung	179	There was no relation between BMI and satisfaction	VAS (0–10)	2011	Australia	Case–control	9.2 years	535	230	305	71.0	28.0	OA	NA	NA	NA	No	NA
Zha	180	No difference between patients with lateral retinacular release and those without it	4 grades (very satisfied, satisfied, unsure, dissatisfied)	2014	China	Randomised controlled trial	18 months	139	46	93	68.2	24.0	OA	MPP	No	Gemini MK II (mobile bearing)	Yes	General
Zha	181	Chondromalacia patellae did not influence satisfaction	4 grades (very satisfied, satisfied, unsure or dissatisfied)	2017	China	Case series	36 months	290	123	167	67.7	25.0	OA	MPP	No	LCS mobile bearing	Yes	NA

Age are shown in years (mean). Body mass index are shown in kg/m^2^ (mean). Full information of the studies are listed in Electronic Supplementary Material 1

From all these studies, we found 98 factors, which could potentially affect patient satisfaction and these were then categorised into seven groups as follows:

1. Patient demographics
2. Non-knee factors
3. Knee factors
4. Factors relating to implants/prostheses
5. Intra-operative technical factors
6. Post-operative outcome factors
7. Surgeon and healthcare factors

**Fig. 2:**
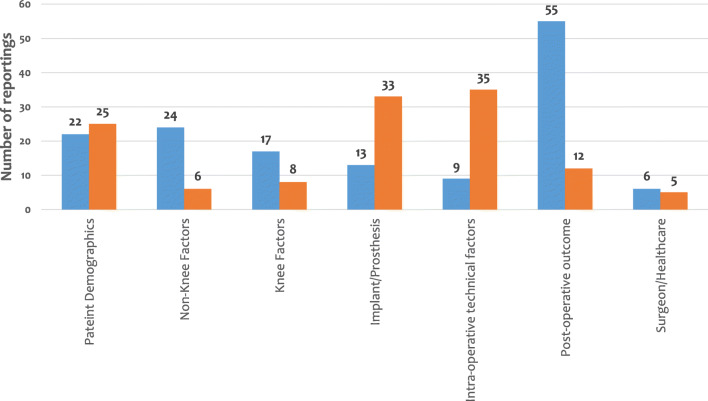
Number of reportings in seven groups of factors for patient satisfaction following total knee replacement. Blue bar means FACTOR (‘it is a factor for patients’ satisfaction’) and orange bar means Not-FACTOR (‘it is a factor which does NOT relate to patients’ satisfaction’)

All the 98 factors as well as scales/scores which were reported to relate to patient satisfaction are summarised in Table 4. Details of the results in each group are described in Electronic Supplementary Material 3. The number of reportings for each group is presented in Fig. 2, and the methods used to measure satisfaction are shown in Table 5.

**Table 4:** Potential factors for patient satisfaction following primary total knee replacement (TKR) with their groups

Factors	Sub-factors for satisfaction	Serial number of reporting studies
1. Patient demographics (47)		
Age (17)	Young	95, 118, 137, 149 (4)
	Old	9, 112, 125, 172 (4)
	Not-FACTOR	1, 7, 40, 47, 72, 126, 147, 158, 176 (9)
Gender (10)	Male	9, 14, 133, 149 (4)
	Female	147 (1)
	Not-FACTOR	7, 40, 72, 126, 176 (5)
Body mass index (BMI), weight (12)	Normal BMI	10, 23, 62, 98, 125 (5)
	Not-FACTOR	7, 47, 72, 126, 162, 176, 179 (7)
Ethnicity (2)	Caucasian > African American	71 (1)
	Not-FACTOR	14 (1)
Income (2)	Annual income > 25,000 USD	14, 133 (2)
Social background (education, employment, insurance) (4)	High education	62 (1)
	Not-FACTOR	14, 80, 133 (3)
2. Non-knee factors (30)		
Back pain (3)	No low back pain	34, 42, 153 (3)
Allergy (2)	No allergy	121, 132 (2)
Fibromyalgia (1)	No fibromyalgia	15 (1)
Problems in other joints (2)	No problem in other joints	117, 153 (2)
General condition (1)	ASA 2 or worse	9 (1)
Comorbidity (1)	No medical comorbidity	7 (1)
Use of narcotics (1)	No use of narcotics	51 (1)
Diabetes mellitus (1)	Not-FACTOR	35 (1)
Generalised joint laxity (1)	Not-FACTOR	102 (1)
Mental health anxiety, depression and personality traits (15)	No mental problem	3, 5, 7, 16, 17, 23, 33, 47,49, 54, 55, 125, 153 (13)
	Not-FACTOR	94, 142 (2)
Pre-operative activity level (2)	Not-FACTOR	128, 171 (2)
3. Knee factors (25)		
Pre-operative stiff knee (1)	No stiff knee	156 (1)
Pre-operative knee pain (4)	No pain at rest	22, 73 (2)
	Chronic pain	149 (1)
	No movement-elicited pain	73 (1)
History of past knee surgery (ACL reconstruction, HTO) (1)	Not-FACTOR	106 (1)
Satisfaction on the first side (in bilateral TKR) (1)	Not-FACTOR	154 (1)
Diagnosis (7)	RA > OA	24 (1)
	Not OA	147 (1)
	RA > OA > post-trauma > AVN	149 (1)
	Not-FACTOR	7, 56, 155, 176 (4)
Degree of degeneration (4)	Severe pre-operative radiographic degenerative change	71, 79, 125, 151 (4)
Chondromalacia patellae (1)	Not-FACTOR	181 (1)
Patellar congruence (1)	Not-FACTOR	157 (1)
Intact ACL in CR-TKR (1)	No intact ACL	70 (1)
Knee extensor strength (1)	Great knee extensor strength	53 (1)
Intra-operative joint force (1)	Greater intra-operative force in the medial compartment	74 (1)
Intra-operative kinematic pattern of the knee (1)	Medial pivot kinematic pattern	136 (1)
Patient’s perspective (1)	High flexion activities	89 (1)
4. Factors related to implants/prostheses (46)		
Specific prosthesis (7)	Triathlon > Kinemax	60 (1)
	Triathlon > Kinemax Plus	46 (1)
	PFC > CKS	123 (1)
	Vega, Genesis II > E.motion	75 (1)
	NexGen > AGC	114 (1)
	Not-FACTOR	81, 145 (2)
Cruciate-retaining/posterior-stabilised/ultra-congruent design (8)	PS > CR	178 (1)
	Not-FACTOR	14, 18, 44, 78, 115, 141, 165 (7)
Design of the bearing (insert) (12)	Mobile-bearing insert	32, 82, 91, 110 (4)
	Rotating mobile > floating mobile	83 (1)
	Not-FACTOR	14, 63, 122, 133, 139, 144, 177 (7)
Single radius prosthesis/multi-radius prosthesis (2)	Single radius > multi-radius	43 (1)
	Not-FACTOR	65 (1)
Use/type/number of stem (1)	Not-FACTOR	12 (1)
Highly cross-linked polyethylene (1)	Not-FACTOR	93 (1)
Material of femoral components (1)	Not-FACTOR	67 (1)
Gender-specific design (6)	Not-FACTOR	14, 84, 88, 133, 139, 166 (6)
High-flexion design (7)	Not-FACTOR	14, 20, 31, 87, 133, 139, 168 (7)
Customised prosthesis (1)	Non-customised (= off-the-shelf) prosthesis	175 (1)
5. Intra-operative technical factors (44)		
Approach, incision (4)	Lateral subvastus approach	66 (1)
	Not-FACTOR	6, 9, 160 (3)
Cement technique (4)	Surface-cemented > fully cemented (for tibial component)	150 (1)
	Not-FACTOR	52, 92, 140 (3)
Kinematic alignment technique (1)	Not-FACTOR	133 (1)
Gap balancing/measured resection technique (1)	Not-FACTOR	41 (1)
Navigation/patient-specific instrument/custom cutting guide/robotic surgery (13)	Using a navigation system	104 (1)
	Not-FACTOR	19, 39, 61, 96, 97, 99, 108, 109, 131, 133, 134, 161 (12)
Patellar resurfacing (13)	Patellar resurfacing	120, 148, 149, 174 (4)
	Not-FACTOR	4, 8, 13, 25, 26, 27, 69, 85, 114 (9)
Lateral retinacular release (1)	Not-FACTOR	180 (1)
Minimally invasive surgery (MIS) (1)	Not-FACTOR	64 (1)
Periarticular injection with corticosteroid (1)	Not-FACTOR	103 (1)
Patellar treatment (in cases without patellar resurfacing) (2)	Patellar denervation	143 (1)
	Patelloplasty	163 (1)
Use of a tourniquet (1)	Not-FACTOR	9 (1)
Removal of fat pad (1)	Not-FACTOR	9 (1)
One-stage/two-stage bilateral TKR (1)	Not-FACTOR	116 (1)
6. Post-operative outcome factors (55)		
Knee alignment (1)	Good post-operative alignment	118 (1)
Pain (8)	No/less pain	2, 3, 7, 9, 29, 48, 170 (7)
	No neuropathic pain	146 (1)
Range of motion (9)	Improvement in ROM	3, 47, 59, 72, 86, 176 (6)
	Not-FACTOR	45, 126, 135 (3)
Flexion contracture (2)	No flexion contracture	32, 57 (2)
Knee swelling (1)	No knee swelling	47 (1)
Radiologic leg length discrepancy (2)	Not-FACTOR	30, 90 (2)
Perception of leg length discrepancy (1)	No perception of leg length discrepancy	30 (1)
Malpositioning of femoral component (4)	Accurate coronal alignment	68 (1)
	Medial malpositioned femoral component (more than 5 mm)	169 (1)
	Accurate rotation	77 (1)
	Not-FACTOR	129 (1)
Malpositioning of tibial component (1)	Not-FACTOR	77 (1)
Residual symptom (1)	No residual symptoms	137 (1)
Physical function (7)	Good physical function	7, 11, 36, 48, 117, 130, 137 (7)
Degree of expectation met (5)	Pre-operative expectations met	21, 22, 40, 137, 164 (5)
Anterior–posterior knee stability (1)	Not-FACTOR	152 (1)
Ligament balance (3)	Good ligament balance of the knee	58, 76, 119 (3)
Medial joint laxity (1)	No medial joint laxity	167 (1)
Lateral joint laxity (1)	Not-FACTOR	167 (1)
Noise (2)	Not-FACTOR	100, 159 (2)
Altered sensation (2)	No numbness	159 (1)
	Not-FACTOR	127 (1)
Complication (3)	No complication	22, 125 (2)
	No deep prosthetic infection	5 (1)
7. Surgeon and healthcare factors (11)		
Type of analgesia used (1)	Not-FACTOR	173 (1)
Post-operative irrigation (1)	Continuous irrigation by cold saline with epinephrine	105 (1)
Post-operative rehabilitation (2)	Patients’ high motivation	47 (1)
	Regular physical activity	28 (1)
Length of hospital stay (2)	Short hospital stay	5 (1)
	Not-FACTOR	176 (1)
Waiting time before TKR (1)	Shorter than 6 months	111 (1)
Country where TKR is conducted (1)	Not-FACTOR	107 (1)
Surgeon’s job title (consultant or not) (1)	Not-FACTOR	9 (1)
Surgeon’s perspective towards the TKR (surgeon’s satisfaction) (1)	Not-FACTOR	124 (1)
Hospital choice (1)	Patients having a hospital choice	113 (1)
(Relating scores/scales) (17)		
Relation (+)	WOMAC score	86, 126, 138, 164 (4)
	Oxford Knee Score	37, 38, 68, 153, 156, 176 (6)
	Knee Society Score	72, 176 (2)
	SF-12 score	153, 176 (2)
	SF-36 score	164 (1)
	Control Preference Scale	50 (1)
Relation (−)	New Knee Society Score	101 (1)

Reporting studies are described using serial numbers in Table 3. The number of each category is shown in parentheses

*Not-FACTOR* ‘it is a factor which does NOT relate to patient satisfaction’

**Table 5:** Measuring methods for patients’ satisfaction

2 Grades (satisfied or not) (15)	
12, 13, 14, 48, 52, 54, 69, 74, 81, 98, 133, 134, 139, 142, 147	
3 Grades (5)	
9, 70, 71, 73, 121	
4 Grades (45)	
2, 3, 4, 5, 10, 15, 29, 33, 34, 35, 36, 37, 38, 39, 40, 46, 57, 59, 60, 72, 83, 86, 89, 91, 107, 113, 115, 116, 117, 120, 126, 143, 149, 153, 154, 155, 156, 157, 161, 173, 174, 176, 177, 180, 181	
5 Grades (36)	
7, 11, 16, 17, 18, 20, 21, 22, 30, 31, 41, 43, 45, 47, 49, 56, 58, 61, 62, 68, 85, 90, 94, 95, 104, 111, 125, 135, 138, 148, 150, 151, 159, 162, 164, 171	
6 Grades (6)	
6, 19, 106, 108, 109, 146	
Numerical Rating Scale (NRS) (0–10) (8)	
50, 63, 79, 80, 129, 158, 169, 172	
Visual Analogue Scale (VAS) (0–10) (28)	
23, 28, 42, 64, 65, 66, 84, 87, 88, 96, 102, 103, 105, 110, 112, 122, 127, 128, 140, 144, 145, 152, 165, 166, 168, 170, 175, 179	
VAS (0–100) (11)	
8, 24, 55, 82, 92, 93, 97, 114, 123, 124, 178	
New Knee Society Score (15)	
32, 53, 76, 77, 78, 100, 101, 118, 119, 130, 131, 132, 136, 160, 167	
British Orthopaedic Association grading system (4)	
1, 67, 75, 127	
Total Knee Function Questionnaire (3)	
44, 137, 141	
Authors’ original questionnaire (5)	
25, 26, 27, 99, 163	
Unclear (1)	
51	

Studies are described using serial numbers in Table 3. The number of studies in each group is shown in parentheses

**Fig. 3:**
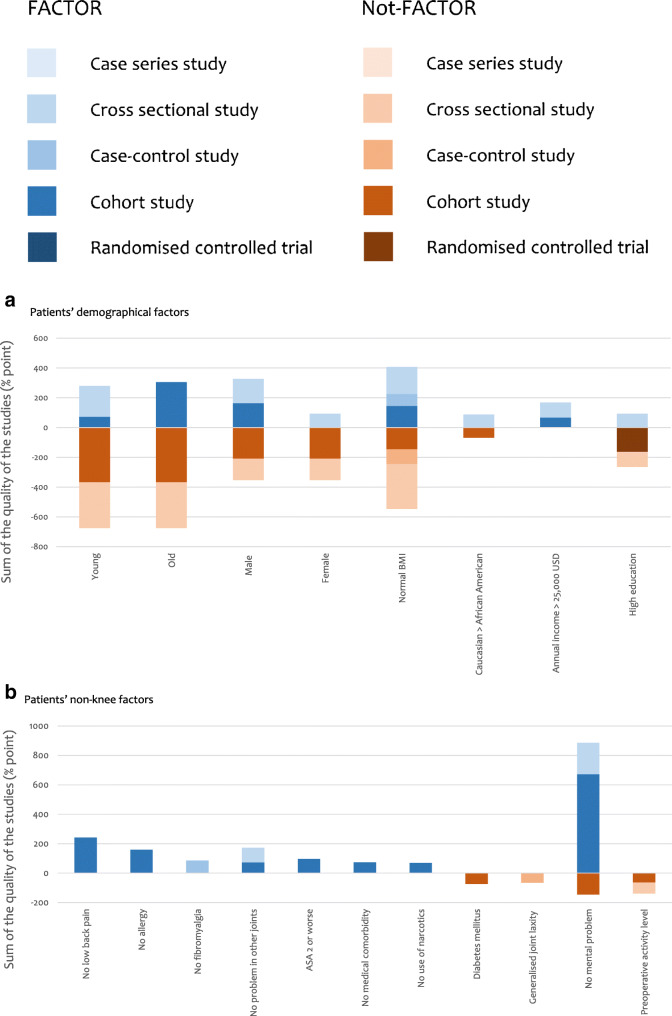

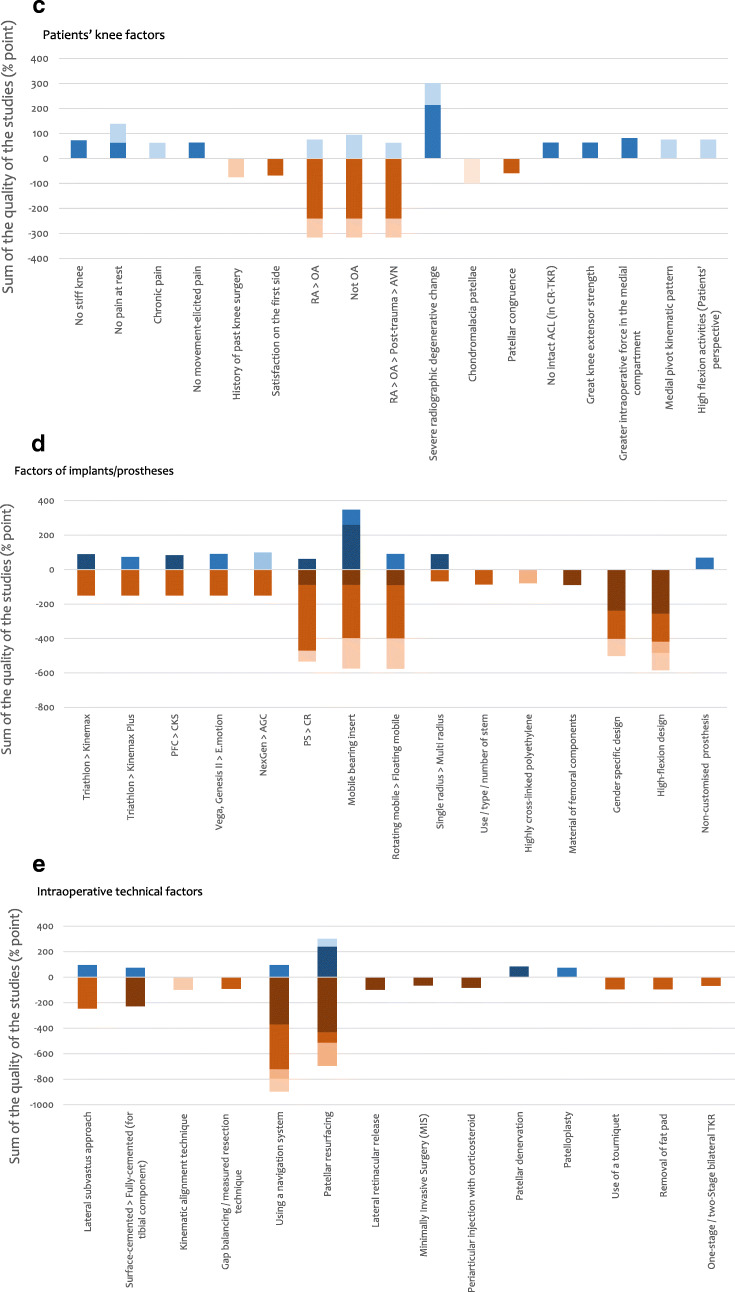

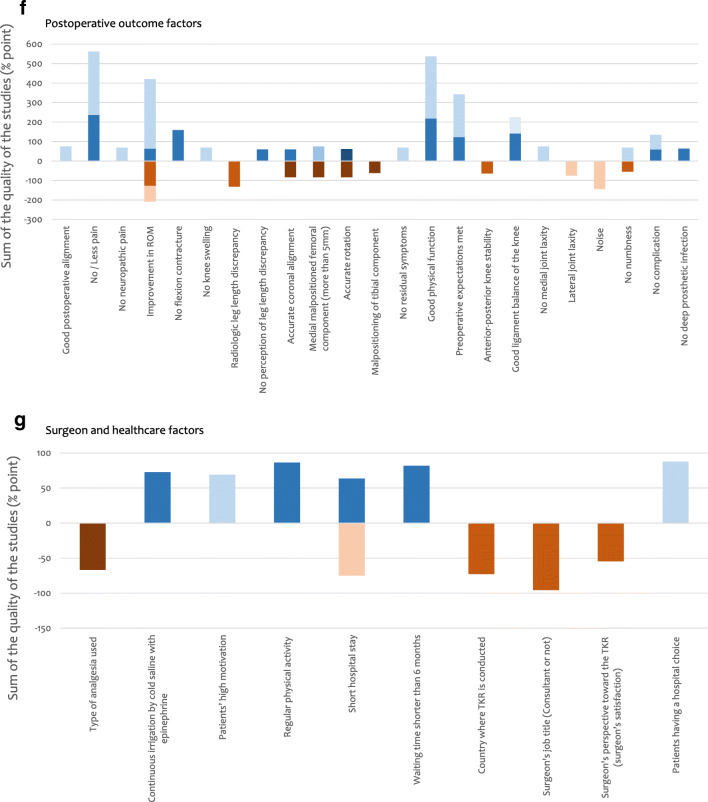
Sum of percentage from full score (%) based on the quality assessment in each type of study for each factor. Blue bar means FACTOR (‘it is a factor for patients’ satisfaction’) and orange bar means Not-FACTOR (‘it is a factor which does NOT relate to patients’ satisfaction’). **a** Patients’ demographical factors. **b** Patients’ non-knee factors. **c** Patients’ knee factors. **d** Factors of implants/prostheses. **e** Intra-operative technical factors. **f** Post-operative outcome factors. **g** Surgeon and healthcare factors

The quality of all the 181 studies was assessed and the results are shown in Tables 6, 7, 8, 9 and 10. The strength of each factor was described using the sum of percentage in each type of study (RCT, cohort study, case–control study, cross-sectional study and case series) (Fig. 3). RCTs were considered to be the strongest (deep colour in Fig. 3) and this was followed by cohort study, case–control study and cross-sectional study, respectively. Case series was considered to be the weakest (light colour in Fig. 3).

**Table 6:** Results of quality assessment of 181 studies—cohort studies: 93 studies. The Joanna Briggs Institute Critical Appraisal Checklist is used

Scoring: Yes = 2 / Unclear = 1 / No = 0 / NA = not applicable Q1: Were the two groups similar and recruited from the same population? Q2: Were the exposures measured similarly to assign people to both exposed and unexposed groups? Q3: Was the exposure measured in a valid and reliable way? Q4: Were confounding factors identified? Q5: Were strategies to deal with confounding factors stated? Q6: Were the groups/participants free of the outcome at the start of the study (or at the moment of exposure)? Q7: Were the outcomes measured in a valid and reliable way? Q8: Was the follow-up time reported and sufficient to be long enough for outcomes to occur? Q9: Was follow-up complete, and if not, were the reasons to lose to follow-up described and explored? Q10: Were strategies to address incomplete follow-up utilised? Q11: Was appropriate statistical analysis used?													
Study (serial no.)	Q1	Q2	Q3	Q4	Q5	Q6	Q7	Q8	Q9	Q10	Q11	Total ( /22)	%
1	2	2	2	1	1	2	2	2	2	1	2	19	86.4
5	1	1	2	0	0	0	2	2	2	2	2	14	63.6
6	2	2	1	1	0	2	2	2	2	2	2	18	81.8
7	0	2	2	0	0	2	2	2	2	2	2	16	72.7
10	2	2	2	2	2	1	2	2	2	2	2	21	95.5
11	0	1	2	0	0	2	2	2	2	2	1	14	63.6
12	2	2	2	0	2	2	2	2	2	1	2	19	86.4
14	0	1	1	1	1	2	2	2	2	1	2	15	68.2
16	0	0	2	0	0	1	2	2	2	2	1	12	54.5
17	0	0	2	0	0	1	2	2	2	2	2	13	59.1
18	2	2	2	0	0	2	2	2	2	1	1	16	72.7
23	1	1	2	2	2	2	2	2	2	1	2	19	86.4
28	0	2	2	2	2	2	2	2	2	1	2	19	86.4
30	0	1	1	0	0	2	2	2	2	1	2	13	59.1
32	2	2	2	1	1	2	2	2	2	1	2	19	86.4
33	0	2	2	2	2	1	2	2	1	0	2	16	72.7
34	2	2	2	2	2	2	2	2	2	0	2	20	90.9
35	0	2	2	2	2	1	2	2	1	0	2	16	72.7
36	2	2	2	1	2	1	2	2	1	1	2	18	81.8
37	0	2	2	0	0	1	2	2	2	0	2	13	59.1
39	2	2	2	0	0	1	2	2	2	0	2	15	68.2
40	0	2	2	0	0	2	2	2	2	0	2	14	63.6
41	2	2	2	2	2	2	2	2	2	0	2	20	90.9
42	2	2	2	0	0	2	2	2	2	2	1	17	77.3
44	2	2	2	2	1	2	2	2	2	0	2	19	86.4
46	2	2	2	0	0	2	2	2	2	0	2	16	72.7
49	0	2	2	1	1	2	2	2	2	1	1	16	72.7
50	0	2	2	0	0	1	2	2	1	1	2	13	59.1
51	2	2	2	0	0	2	2	2	1	0	2	15	68.2
53	0	2	2	0	0	2	2	2	2	0	2	14	63.6
54	0	2	2	0	0	2	2	2	1	0	2	13	59.1
56	2	2	2	2	2	2	2	2	2	0	2	20	90.9
57	2	2	2	0	0	2	2	2	2	0	2	16	72.7
58	2	2	2	0	0	2	2	2	1	0	2	15	68.2
59	0	2	2	0	0	2	2	2	2	0	2	14	63.6
65	2	1	2	0	0	2	2	2	2	0	2	15	68.2
66	2	2	2	2	2	2	2	2	2	1	2	21	95.5
68	0	2	2	0	0	2	2	2	1	0	2	13	59.1
70	0	2	2	1	0	2	2	2	1	0	2	14	63.6
73	0	2	2	0	0	2	2	2	2	0	2	14	63.6
74	2	2	2	1	2	2	2	2	2	0	1	18	81.8
75	2	2	2	2	2	2	2	2	2	0	2	20	90.9
79	2	2	2	2	2	2	2	2	2	1	2	21	95.5
80	2	2	2	2	2	2	2	2	2	1	2	21	95.5
81	2	2	2	0	0	2	2	1	0	0	1	12	54.5
83	2	2	2	2	2	2	2	2	2	0	2	20	90.9
85	2	2	2	1	1	2	2	2	2	0	2	18	81.8
90	2	2	2	0	0	2	2	2	2	0	2	16	72.7
94	2	2	2	0	0	2	2	2	2	0	2	16	72.7
95	2	2	2	0	0	2	2	2	2	0	2	16	72.7
99	2	2	2	0	0	2	2	2	2	0	2	16	72.7
101	0	2	2	0	0	2	2	2	1	0	2	13	59.1
104	2	2	2	2	1	2	2	2	2	2	2	21	95.5
105	2	2	2	0	0	2	2	2	2	0	2	16	72.7
107	0	2	1	1	2	2	2	2	2	0	2	16	72.7
111	2	2	2	0	1	2	2	2	2	1	2	18	81.8
112	1	2	2	0	0	2	2	2	2	0	2	15	68.2
115	2	2	2	2	2	2	2	2	1	0	2	19	86.4
116	2	2	2	0	0	2	2	2	1	0	2	15	68.2
119	0	2	2	1	1	2	2	2	2	0	2	16	72.7
121	0	2	2	2	2	2	2	2	1	0	2	17	77.3
122	2	2	2	0	0	2	2	2	1	0	2	15	68.2
124	0	1	2	0	0	2	2	2	1	0	2	12	54.5
125	0	2	1	0	0	2	2	2	2	0	2	13	59.1
126	1	1	2	1	2	2	2	2	1	0	2	16	72.7
127	1	2	2	0	0	2	2	1	1	0	1	12	54.5
128	2	2	2	0	0	2	2	2	1	0	1	14	63.6
131	2	2	2	0	0	2	2	2	2	0	1	15	68.2
132	0	2	2	2	2	2	2	2	2	1	1	18	81.8
134	1	2	2	0	0	2	2	2	2	0	1	14	63.6
135	0	2	2	0	0	2	2	2	1	0	1	12	54.5
138	1	2	2	2	1	2	2	2	2	0	2	18	81.8
139	2	2	2	2	2	2	2	2	2	1	2	21	95.5
141	2	2	2	0	0	2	2	2	1	0	2	15	68.2
142	2	2	2	0	0	2	2	2	2	0	2	16	72.7
144	2	2	2	1	0	2	2	2	2	0	2	17	77.3
145	2	2	2	2	1	2	2	2	2	2	2	21	95.5
150	2	2	2	0	0	2	2	2	2	0	2	16	72.7
151	0	2	2	0	0	2	2	2	1	0	2	13	59.1
152	0	2	2	0	0	2	2	2	2	0	2	14	63.6
153	0	2	2	1	1	2	2	2	2	0	2	16	72.7
154	0	2	2	0	0	2	2	2	2	1	2	15	68.2
155	1	2	2	1	1	2	2	2	2	0	2	17	77.3
156	0	2	2	1	1	2	2	2	2	0	2	16	72.7
157	0	2	2	0	0	2	2	2	1	0	2	13	59.1
158	2	2	2	0	0	2	2	2	2	0	2	16	72.7
160	2	2	2	0	0	2	2	2	1	0	2	15	68.2
161	2	2	2	0	1	2	2	2	2	0	2	17	77.3
163	2	2	2	0	0	2	2	2	2	0	2	16	72.7
164	0	2	2	0	0	2	2	2	1	0	2	13	59.1
170	0	2	2	1	1	2	2	2	1	0	2	15	68.2
172	2	2	2	1	1	2	2	2	2	0	2	18	81.8
175	2	2	2	0	0	2	2	2	2	0	1	15	68.2

Studies are described using serial numbers in Table 3

**Table 7:** Results of quality assessment of 181 studies—case–control studies: 9 studies. The Joanna Briggs Institute Critical Appraisal Checklist is used

Scoring: Yes = 2 / Unclear = 1 / No = 0 / NA = not applicableQ1: Were the groups comparable other than the presence of disease in cases or the absence of disease in controls? Q2: Were cases and controls matched appropriately? Q3: Were the same criteria used for identification of cases and controls? Q4: Was exposure measured in a standard, valid and reliable way? Q5: Was exposure measured in the same way for cases and controls? Q6: Were confounding factors identified? Q7: Were strategies to deal with confounding factors stated? Q8: Were outcomes assessed in a standard, valid and reliable way for cases and controls? Q9: Was the exposure period of interest long enough to be meaningful? Q10: Was appropriate statistical analysis used?												
Study (serial no.)	Q1	1. Q2	2. Q3	3. Q4	4. Q5	5. Q6	6. Q7	7. Q8	8. Q9	9. Q10	Total ( /20)	%
15	2	1	2	2	2	1	1	2	2	2	17	85.0
20	1	1	1	2	2	1	1	2	0	2	13	65.0
69	1	1	1	2	2	2	1	2	2	2	16	80.0
93	2	2	2	2	2	0	0	2	2	2	16	80.0
98	2	2	2	2	2	0	0	2	2	2	16	80.0
102	1	1	1	2	2	0	0	2	2	2	13	65.0
108	2	1	1	2	2	1	1	2	1	2	15	75.0
114	2	2	2	2	2	2	2	2	2	2	20	100.0
179	2	2	2	2	2	2	2	2	2	2	20	100.0

Studies are described using Serial numbers in Table 3

**Table 8:** Results of quality assessment of 181 studies—cross-sectional studies: 37 studies. The Joanna Briggs Institute Critical Appraisal Checklist is used

Scoring: Yes = 2 / Unclear = 1 / No = 0 / NA = not applicable Q1: Were the criteria for inclusion in the sample clearly defined? Q2: Were the study subjects and the setting described in detail? Q3: Was the exposure measured in a valid and reliable way? Q4: Were objective, standard criteria used for measurement of the condition? Q5: Were confounding factors identified? Q6: Were strategies to deal with confounding factors stated? Q7: Were the outcomes measured in a valid and reliable way? Q8: Was appropriate statistical analysis used?										
Study (serial no.)	Q1	Q2	Q3	Q4	Q5	Q6	Q7	Q8	Total ( /16)	%
2	2	2	2	2	1	1	2	2	14	87.5
3	2	2	1	2	0	0	2	2	11	68.8
9	2	2	2	2	1	1	2	2	14	87.5
21	2	2	2	2	0	0	2	2	12	75.0
22	2	2	2	2	0	0	2	2	12	75.0
24	2	2	2	2	0	0	2	2	12	75.0
29	1	2	2	2	2	2	2	2	15	93.8
38	2	2	2	2	2	2	2	2	16	100.0
45	1	2	2	2	1	1	2	2	13	81.3
47	1	2	2	2	0	0	2	2	11	68.8
48	2	2	2	2	0	0	2	2	12	75.0
55	2	2	2	2	0	0	2	2	12	75.0
62	2	2	2	2	1	2	2	2	15	93.8
63	1	2	2	1	1	1	2	2	12	75.0
71	1	2	2	2	1	2	2	2	14	87.5
72	1	2	2	2	0	0	2	2	11	68.8
77	1	1	2	2	0	0	2	2	10	62.5
86	2	2	2	2	0	0	2	2	12	75.0
89	2	2	2	2	0	0	2	2	12	75.0
100	2	2	2	2	0	0	2	2	12	75.0
106	2	2	2	2	0	0	2	2	12	75.0
113	2	2	2	2	0	2	2	2	14	87.5
117	2	2	2	2	2	2	2	2	16	100.0
118	2	2	2	2	0	0	2	2	12	75.0
130	2	2	2	2	0	0	2	2	12	75.0
133	2	2	2	2	2	2	2	2	16	100.0
136	2	2	2	2	0	0	2	2	12	75.0
137	1	2	2	2	0	0	2	2	11	68.8
146	1	2	2	2	0	0	2	2	11	68.8
147	2	2	2	2	1	2	2	2	15	93.8
149	0	2	2	2	0	0	2	2	10	62.5
159	1	2	2	2	0	0	2	2	11	68.8
162	1	2	2	2	1	2	2	2	14	87.5
167	2	2	2	2	0	0	2	2	12	75.0
169	2	2	2	2	0	0	2	2	12	75.0
171	2	2	2	2	0	0	2	2	12	75.0
176	2	2	2	2	0	0	2	2	12	75.0

Studies are described using serial numbers in Table 3

**Table 9:** Results of quality assessment of 181 studies—case series studies: 2 studies. The Joanna Briggs Institute Critical Appraisal Checklist is used

Scoring: Yes = 2 / Unclear = 1 / No = 0 / NA = not applicable Q1: Were there clear criteria for inclusion in the case series? Q2: Was the condition measured in a standard, reliable way for all participants included in the case series? Q3: Were valid methods used for identification of the condition for all participants included in the case series? Q4: Did the case series have consecutive inclusion of participants? Q5: Did the case series have complete inclusion of participants? Q6: Was there clear reporting of the demographics of the participants in the study? Q7: Was there clear reporting of clinical information of the participants? Q8: Were the outcomes or follow-up results of cases clearly reported? Q9: Was there clear reporting of the presenting site(s)/clinic(s) demographic information? Q10: Was statistical analysis appropriate?												
Study (serial no.)	Q1	1. Q2	1. Q3	1. Q4	1. Q5	1. Q6	1. Q7	1. Q8	1. Q9	10. Q10	Total ( /20)	%
76	1. 2	2. 2	2. 1	2. 1	1. 2	1. 2	1. 2	1. 1	2. 2	11. 2	17	85.0
181	2. 2	3. 2	3. 2	3. 2	2. 2	2. 2	2. 2	2. 2	3. 2	12. 2	20	100.0

Studies are described using serial numbers in Table 3

**Table 10:** Results of quality assessment of 181 studies—randomised controlled trials: 40 studies. A modified version of critical appraisal checklist by van Tulder et al [15] is used

Scoring: Yes = 2 / Unclear = 1 / No = 0 / NA = not applicable Q1: Acceptable method of randomisation Q2: Concealed treatment allocation Q3: Similar group values at baseline Q4: Blinded assessor Q5: No or similar co-interventions Q6: Acceptable compliance (≥ 75%) Q7: Acceptable drop-out rate (≤ 30%) Q8: Similar timing of the outcome assessment in all groups Q9: Intention to treat analysis											
Study (serial no.)	Q1	Q2	Q3	Q4	Q5	Q6	Q7	Q8	Q9	Total ( /18)	%
4	2	2	2	2	2	2	2	2	0	16	88.9
8	2	2	2	2	2	2	2	2	2	18	100.0
13	2	2	2	2	2	2	2	2	0	16	88.9
19	2	2	2	2	2	2	2	2	0	16	88.9
25	2	2	2	2	2	2	0	2	0	14	77.8
26	2	2	2	2	2	1	1	2	0	14	77.8
27	2	2	2	2	2	1	0	2	0	13	72.2
31	2	1	2	2	2	2	2	2	2	17	94.4
43	2	2	2	2	2	2	2	2	0	16	88.9
52	1	1	2	0	2	2	2	2	0	12	66.7
60	2	2	2	2	2	2	2	2	0	16	88.9
61	1	1	2	2	2	1	0	2	0	11	61.1
64	1	1	2	0	2	2	2	2	0	12	66.7
67	2	2	2	2	2	2	2	2	0	16	88.9
78	1	1	1	0	2	2	2	2	0	11	61.1
82	2	2	2	2	2	2	2	2	0	16	88.9
84	2	2	2	0	2	2	2	2	0	14	77.8
87	2	1	2	1	2	2	2	2	0	14	77.8
88	1	1	2	2	2	2	2	2	0	14	77.8
91	2	2	2	2	2	2	2	2	0	16	88.9
92	2	2	2	0	2	2	2	2	0	14	77.8
96	2	1	2	0	2	2	2	2	2	15	83.3
97	2	1	2	0	2	2	2	2	0	13	72.2
103	2	1	2	2	2	2	2	2	0	15	83.3
109	2	0	2	0	2	2	2	2	0	12	66.7
110	2	1	2	2	2	2	2	2	0	15	83.3
120	1	1	2	2	2	2	2	2	0	14	77.8
123	2	1	2	2	2	2	2	2	0	15	83.3
129	2	1	2	2	2	2	2	2	0	15	83.3
140	2	1	2	2	2	2	2	2	0	15	83.3
143	2	1	2	2	2	2	2	2	0	15	83.3
148	2	2	2	2	2	2	2	2	0	16	88.9
165	2	2	2	2	2	2	2	2	0	16	88.9
166	2	1	2	2	2	2	2	2	0	15	83.3
168	2	1	2	2	2	2	2	2	0	15	83.3
173	1	1	2	2	2	1	1	2	0	12	66.7
174	2	1	2	2	2	1	1	2	0	13	72.2
177	2	2	2	2	2	2	2	2	0	16	88.9
178	1	1	2	1	2	1	1	2	0	11	61.1
180	2	2	2	2	2	2	2	2	2	18	100.0

Studies are described using serial numbers in Table 3

When the results of the quality assessment were taken into consideration, a negative history of mental health problems, use of a mobile-bearing insert, patellar resurfacing, severe pre-operative radiological degenerative change, negative history of low back pain, no/less post-operative pain, good post-operative physical function and pre-operative expectations being met were considered to be important factors. Significant factors affecting patient satisfaction are summarised in Tables 11, 12 and 13.

**Table 11:** List of frequently reported factors as FACTOR (‘it is a factor for patient satisfaction’)

	Factors (number of reportings)
1st place	No mental health problems (13 reportings)
2nd place	No/less post-operative pain (7 reportings)
2nd place	Good post-operative physical function (7 reportings)
4th place	Improvement in ROM (6 reportings)
5th place	Normal BMI (5 reportings)
5th place	Pre-operative expectations met (5 reportings)

*BMI* body mass index, *ROM* range of motion

**Table 12:** List of factors which have the highest sum of percentage score (a percentage from full score) of FACTOR (‘it is a factor for patient satisfaction’) only based on the quality assessment for various combination of the types of the studies

	RCT	RCT + Cohort	RCT + Cohort + Case–control	RCT + Cohort + Case–control + Cross-sectional	RCT + Cohort + Case–control + Cross-sectional + Case series
1st place	Use of mobile bearing insert (261.1%)	No mental health problems (672.6%)	No mental health problems (672.6%)	No mental health problems (885.2%)	No mental health problems (885.2%)
2nd place	Patellar resurfacing (238.9 %)	Use of mobile-bearing insert (347.5%)	Use of mobile bearing insert (347.5%)	No/less post-operative pain (561.5%)	No/less post-operative pain (561.5%)

*RCT* randomised controlled trial

**Table 13:** List of factors which have the highest sum of percentage score (a percentage from full score) of FACTOR (‘it is a factor for patient satisfaction’) and Not-FACTOR (‘it is a factor which does NOT relate to patient satisfaction’) based on the quality assessment for all type of the studies

	Factors (% score)
1st place	No mental health problems (739.8%)
2nd place	No/less post-operative pain (561.5%)
3rd place	Good physical function (536.9%)
4th place	Pre-operative expectations met (341.5%)
5th place	Severe pre-operative radiographic degenerative change (301.2%)
6th place	No low back pain (240.9%)

Percentage score of Not-FACTOR was calculated as negative value

## Discussion

The dissatisfaction rate following a TKR remains around 20% and is a constant source of frustration for the patient and the surgeon [11, 12]. Our study has systematically reviewed all the articles looking at satisfaction following a TKR to determine the factors, which could be responsible for this issue. Several factors were deemed to be important in affecting patient satisfaction based on the number of studies in which they were reported as well as the results of the quality assessment of the study (Tables 11, 12 and 13).

### Negative history of mental health problems

A negative history of mental health problems was the most frequently reported factor affecting patient satisfaction (Table 11) and also scored the highest sum of percentage of FACTOR based on the quality assessment for RCT + cohort study (± case–control study ± cross-sectional study ± case series study) (Table 12). In addition, it was ranked first in terms of the highest sum of percentage of FACTOR and Not-FACTOR based on the quality assessment for all types of the studies (Table 13). Depressive symptoms and anxiety were reported to be predictive of long-term pain and functional impairment as measured by the Knee Society Score in 83 patients at 5 years [16]. In addition, it was reported that pre-operative anxiety/depression is an independent risk for severe post-operative pain and may explain as to why there is a subset of patients with unexplained pain after surgery [17]. Moreover, Macleod et al. report that patients with mental disability suffered a greater level of comorbidity and were socially deprived, which is also related to poorer physical health which then has an impact on satisfaction [18]. Finally, another study reported that patients with poor mental health, which can impair coping mechanisms for pain, might present with less severe disease, and this could also influence their satisfaction [19].

### Use of a mobile-bearing insert

The use of a mobile-bearing insert had the highest sum of percentage of FACTOR based on the quality assessment for RCTs. Also, it had the second highest sum of percentage of FACTOR based on the quality assessment for RCT + cohort study (± case–control study) (Table 12). The rationale behind the design of a mobile-bearing insert is to solve the kinematic conflict between low-stress articulation and free axial femoral–tibial rotation by allowing rotation of a highly conforming polyethylene insert [20]. Theoretically, the design of the mobile-bearing insert could lead to better ROM especially during flexion [21]. A greater loss of flexion was reported after 12 months in patients with a TKR with a fixed-bearing prosthesis in comparison with a mobile-bearing prosthesis [22]. It is quite intuitive to comprehend that a good post-operative ROM relates to patient satisfaction, and our results support this (improvement in ROM was the 4th most frequently reported factor for patient satisfaction). Kim et al. suspect the low constraint of mobile-bearing insert may restore normal kinematics of the knee and it contributes to favourable clinical outcomes compared with a fixed-bearing insert [23]. Price et al. in a prospective multicentre trial of 39 simultaneous bilateral procedures also found that patients with a mobile-bearing insert had significantly better clinical results than patients with a fixed-bearing insert [21].

### Patellar resurfacing

Patellar resurfacing has the second highest sum of percentage of FACTOR based on the quality assessment for RCTs (Table 12). Four studies showed patients with patella resurfacing were more satisfied than those without it [11, 24–26]. Amongst them, one study focused on only knees with no exposed bone on the undersurface of the patella to determine the potential advantages of leaving the patella non-resurfaced [25]. Dissatisfaction in patella non-resurfaced patients may be due to the higher rate of post-operative anterior knee pain, and patients whose patella was not resurfaced at the index TKR tended to have a higher revision rate as well [25–28]. However, it should be noted that this issue may be strongly related to the design of the implant. There have also been abundant literature that showed that the patellofemoral design in TKR is critical and can vary the forces on the patellofemoral joint as well as patellofemoral tracking [29–31]. Two of the 4 studies relate to a specific prosthesis (PFC) which is notoriously patella unfriendly [25, 26], so this relationship may therefore not necessarily hold true for the newer implants with patella-friendly designs.

### Severe pre-operative radiological degenerative change

Severe pre-operative radiological degenerative change has the fifth highest sum of percentage of FACTOR and Not-FACTOR based on the quality assessment for all types of studies (Table 13). Although the classic indication for replacing a patient’s knee is end-stage arthritis (Kellgren–Lawrence grade IV [32]), there are a number of patients who have a TKR much before grade IV radiological changes have set in and it is dependent on the symptoms of the patient. The individual indication is complex and involves multiple factors [33]. Patients with mild pre-operative OA were reported to have a worse prognosis in improvement in physical functioning [34, 35], and therefore, it is difficult to meet their expectations post-operatively [35]. These effects are more noticeable in patients undergoing a TKR as compared with those who have had a THR [34]. The knee is a complex joint and the biomechanics of this joint are much more difficult to replicate with a prosthetic knee as compared with a prosthetic hip which may partly explain a smaller increase in physical functioning and a poor rate of satisfaction in patients with mild OA having a TKR [36].

### No low back pain

No low back pain has the sixth highest sum of percentage of FACTOR and Not-FACTOR based on the quality assessment for all types of the studies (Table 13). The prevalence of chronic low back pain in the UK has been reported to range from 6 to 11% [29], and this is increased to 55% in patients with OA of the knee [30]. Furthermore, low back pain has been demonstrated to be three to four times more likely to be present in patients with a history of depression [37]. Also, patients with chronic low back pain have a higher rate of musculoskeletal and neuropathic pain conditions, depression, anxiety and sleep disorders [31]. In addition, patients with low back pain reported to have more symptoms from their osteoarthritic knee which may suggest a lower threshold for pain in this cohort leading to dissatisfaction [30].

### Normal BMI

Normal BMI was the fifth most frequently reported factor for patient satisfaction (Table 11). BMI greater than 30 kg/m^2^ was reported to be associated with a higher rate of revision and poorer functional outcomes as well which again contributes to dissatisfaction [38]. In addition, morbidly obese patients are likely to suffer from wound problems, ligament injuries and infections peri-operatively which lead to dissatisfaction [22]. Another study showed that despite lower pre- and post-operative WOMAC and SF-36 scores, obese patients experienced similar improvements compared with non-obese patients, although levels of satisfaction in the obese group were lower than those in the non-obese group [39]. The authors stated that one explanation for this might be that satisfaction was more closely associated with the absolute post-operative functional level rather than the magnitude of any improvement, as the rate of satisfaction mirrored absolute values of post-operative WOMAC and SF-36 scores.

### Other factors

Other than factors discussed in the previous section, no/less post-operative pain, good post-operative physical function, improvement in ROM and pre-operative expectations being met were considered to be important for patient satisfaction based on the number of reportings and the results of quality assessment (Tables 11, 12 and 13). TKR is a painful procedure and it does take at least six to 12 months to get the maximum benefit from this procedure [40], and therefore, setting realistic expectations with the patient in the pre-operative clinic is essential to avoid dissatisfaction.

## Limitations and strengths of the study

Our study has several limitations. Firstly, the method of measuring satisfaction is different in each study, and therefore, a uniform way of assessing satisfaction is essential for the orthopaedic community. Secondly, the timing of assessment of satisfaction after the index TKR varied amongst studies and this again requires standardisation. Thirdly, in many of the studies included in this review, the authors have only focused on one factor and the mutual or overall effect of multiple factors was not assessed. Fourthly, no statistical tests of intra-class correlation coefficients, inter-rater reliability and heterogeneity amongst the studies were performed in this systematic review. Finally, there are several studies in which patients are duplicated amongst studies and our review was limited to publications in English, so there is a possibility of publication bias.

However, despite all these limitations, the main strength of this study lies in its broad and comprehensive initial literature search as well as complete and in-depth quality assessment for each study and the factors. We have determined all the factors which could potentially affect patient satisfaction following a TKR which have been reported in the literature thus far.

## Conclusion

No history of mental health problems, use of a mobile bearing insert, patellar resurfacing, severe pre-operative radiological degenerative change, no low back pain, normal BMI, no/less post-operative pain, good physical function post-operatively, improvement in ROM and pre-operative expectations being met were considered to be significant factors leading to better patient satisfaction following a TKR.

Surgeons performing a TKR should take these factors into consideration prior to deciding whether a patient is suitable for a TKR. Secondarily, a detailed explanation of these factors should form part of the process of informed consent to achieve better patient satisfaction following TKR.

There is great need for a unified approach to assessing satisfaction following a TKR and also the time at which satisfaction is assessed.

Moreover, further studies and ideally larger RCTs focusing on each of these factors are required to determine the exact correlation of these factors with satisfaction.

## Electronic supplementary material

ESM 1 (DOCX 15.6 kb)

ESM 2 (DOCX 41.7 kb)

ESM 3 (DOCX 17.8 kb)
