# Supplementary material for: Why are patients dissatisfied following a total knee replacement? A systematic review
Source: Int Orthop. 2020 Jul 8;44(10):1971–2007. doi: 10.1007/s00264-020-04607-9 (PMC7584563; doi:10.1007/s00264-020-04607-9)
Supplement: Supplementary file 1 — (DOCX 15.6 kb) [file 264_2020_4607_MOESM1_ESM.docx]

**Appendix 1**

**Search Strategy**

Reference lists of abstracted literature were assessed for additional studies. Database search was conducted to include studies since inception until 31st of December 2017. The research question and individual study eligibility criteria were established a priori. The PICO (participants, interventions, comparators, outcomes) tool was adopted and modified, since no comparators were used in this study, to formulate the research question and establish the inclusion and exclusion criteria. The following search strings were used to identify studies for the databases: Satisfaction, dissatisfaction, TKR, TKA, total knee replacement, total knee arthroplasty and they were all combined with the Boolean terms (Table 1). Articles found were then pooled and exported to Mendeley reference manager software (Elsevier, Amsterdam, Netherlands) and duplicates were removed first, electronically and then manually.

**Data Extraction**

The senior investigator reviewed a random sample (25%) of the excluded studies and all the included title and abstracts for the purpose of quality control. This process led to 100% agreement amongst the three authors. Abstracted data included study-specific general information (author, publication year, country of the study conducted, type of the study), the way to measure patient satisfaction, number of patients/knees, mean age (years), sex distribution, mean body mass index (BMI) (kg/m2), average length of follow-up, primary diagnosis, surgical approach, type of prosthesis, use of navigation/patient specific instrument (PSI), patellar resurfacing, type of bearing, type of anaesthesia, and post-operative pain control. The main outcome measures included any factors/predictors/causes affecting or relating to patient satisfaction following a TKR.

**Data Analysis**

A scoring system was implemented based on the findings from the studies. Both checklists scored the answers to their questions as yes, no, unclear or not applicable. Numerical scoring values were then allocated to each answer where domains answering with yes get 2 points, unclear gets 1 point and no gets 0 point. For easy analysis of the studies, serial numbers were added to name the studies to ensure adequate identification of the studies during the analysis. Some studies were written by the same authors and hence overlapping of patients in studies might have occurred. As a result, authors of all studies, where duplicate cohorts of patients might have been used, were contacted individually to identify those studies and avoid duplicate reporting of factors.
