# Supplementary material for: Why are patients dissatisfied following a total knee replacement? A systematic review
Source: Int Orthop. 2020 Jul 8;44(10):1971–2007. doi: 10.1007/s00264-020-04607-9 (PMC7584563; doi:10.1007/s00264-020-04607-9)
Supplement: Supplementary file 2 — (DOCX 41.7 kb) [file 264_2020_4607_MOESM2_ESM.docx]

**Appendix 2**

181 studies included in the final quality assessment

Serial numbers are the same as those in Table 3

| **Serial Number** | **Study list** |
| --- | --- |
| **Randomised-controlled trials** | |
| 4 | Ali A, Lindstrand A, Nilsdotter A, Sundberg M. Similar patient-reported outcomes and performance after total knee arthroplasty with or without patellar resurfacing. Acta Orthop. 2016;87(3):274-9. |
| 8 | Aunan E, Næss G, Clarke-Jenssen J, Sandvik L, Kibsgard TJ. Patellar resurfacing in total knee arthroplasty: Functional outcome differs with different outcome scores. Acta Orthop. 2016;87(2):158–64. |
| 13 | Barrack RL, Bertot AJ, Wolfe MW, Waldman DA, Milicic M, Myers L. Patellar resurfacing in total knee arthroplasty. A prospective, randomized, double-blind study with five to seven years of follow-up. J Bone Joint Surg Am. 2001;83–A(9):1376–81. |
| 19 | Blyth MJG, Smith JR, Anthony IC, Strict NE, Rowe PJ, Jones BG. Electromagnetic navigation in total knee arthroplasty-A single center, randomized, single-blind study comparing the results with conventional techniques. J Arthroplasty. 2015;30(2):199–205. |
| 25 | Burnett RS, Haydon CM, Rorabeck CH, Bourne RB. The John Insall Award :Patella Resurfacing versus Nonresurfacing in Total Knee Arthroplasty. Clin Orthop Relat Res. 2004;428(428):12–25. |
| 26 | Burnett RSJ, Boone JL, McCarthy KP, Rosenzweig S, Barrack RL. A prospective randomized clinical trial of patellar resurfacing and nonresurfacing in bilateral TKA. Clin Orthop Relat Res. 2007;464(464):65–72. |
| 27 | Burnett RSJ, Boone JL, Rosenzweig SD, Steger-May K, Barrack RL. Patellar resurfacing compared with nonresurfacing in total knee arthroplasty: A concise follow-up of a randomized trial. J Bone Joint Surg Am. 2009;91(11):2562–7. |
| 31 | Choi WC, Lee S, Seong SC, Jung JH, Lee MC. Comparison between standard and high-flexion posterior-stabilized rotating-platform mobile-bearing total knee arthroplasties: A randomized controlled study. J Bone Joint Surg Am. 2010;92(16):2634–42. |
| 43 | Collados-Maestre I, Lizaur-Utrilla A, Gonzalez-Navarro B, Miralles-Muñoz FA, Marco-Gomez L, Lopez-Prats FA, et al. Better functional outcome after single-radius TKA compared with multi-radius TKA. Knee Surg Sports Traumatol Arthrosc. 2017;25(11):3508–14. |
| 52 | Fricka KB, Sritulanondha S, McAsey CJ. To Cement or Not? Two-Year Results of a Prospective, Randomized Study Comparing Cemented Vs. Cementless Total Knee Arthroplasty (TKA). J Arthroplasty [Internet]. 2015/06/30. 2015;30(9):55–8. Available from: http://ac.els-cdn.com/S0883540315004799/1-s2.0-S0883540315004799-main.pdf?_tid=20188380-9c49-11e6-a367-00000aab0f6c&acdnat=1477575036_8bca7a6184bc1bed5f97b3ec56d888fa |
| 60 | Hamilton DF, Burnett R, Patton JT, Howie CR, Moran M, Simpson AHRW, et al. Implant design influences patient outcome after total knee arthroplasty: a prospective double-blind randomised controlled trial. Bone Joint J. 2015;97–B(1):64–70. |
| 61 | Harvie P, Sloan K, Beaver RJ. Computer Navigation vs Conventional Total Knee Arthroplasty. Five-Year Functional Results of a Prospective Randomized Trial. . Vol. 27, Journal of Arthroplasty. 2012;27:667–72. |
| 64 | Hernandez-Vaquero D, Noriega-Fernandez A, Suarez-Vazquez A. Total knee arthroplasties performed with a mini-incision or a standard incision. Similar results at six months follow-up. BMC Musculoskelet Disord. 2010;11:27. |
| 67 | Hui C, Salmon L, Maeno S, Roe J, Walsh W, Pinczewski L. Five-year comparison of oxidized zirconium and cobalt-chromium femoral components in total knee arthroplasty a randomized controlled trial . J Bone Joint Surg Am. 2011;98:624–30. |
| 78 | Kawakami Y, Matsumoto T, Takayama K, Ishida K, Nakano N, Matsushita T, et al. Intermediate-Term Comparison of Posterior Cruciate-Retaining Versus Posterior-Stabilized Total Knee Arthroplasty Using the New Knee Scoring System. Orthopedics [Internet]. 2015/12/15. 2015;38(12):e1127–32. Available from: http://www.healio.com/doiresolver?doi=10.3928/01477447-20151123-03 |
| 82 | Kim YH, Yoon SH, Kim JS. Early outcome of TKA with a medial pivot fixed-bearing prosthesis is worse than with a PFC mobile-bearing prosthesis. Clin Orthop Relat Res. 2009;467(2):493–503. |
| 84 | Kim YH, Choi Y, Kim JS. Comparison of a standard and a gender-specific posterior cruciate-substituting high-flexion knee prosthesis: A prospective, randomized, short-term outcome study. J Bone Joint Surg Am. 2010;92(10):1911–20. |
| 87 | Kim YH, Choi Y, Kim JS. Range of motion of standard and high-flexion posterior cruciate-retaining total knee prostheses: A prospective randomized study. J Bone Jointt Surg Am. 2009;91(8):1874–81. |
| 88 | Kim Y-H, Choi Y, Kim J-S. Comparison of standard and gender-specific posterior-cruciate-retaining high-flexion total knee replacements: A PROSPECTIVE, RANDOMISED STUDY. J Bone Joint Surg Br. 2010;92–B(5):639–45. |
| 91 | Kim YH, Park JW, Kim JS. Clinical Outcome of Medial Pivot Compared With Press-Fit Condylar Sigma Cruciate-Retaining Mobile-Bearing Total Knee Arthroplasty. J Arthroplasty. 2017;32(10):3016–23. |
| 92 | Kim Y-H, Park J-W, Lim H-M, Park E-S. Cementless and cemented total knee arthroplasty in patients younger than fifty five years. Which is better?. International Orthopaedics. 2014;38:297–303. |
| 96 | Kosse NM, Heesterbeek PJC, Schimmel JJP, van Hellemondt GG, Wymenga AB, Defoort KC. Stability and alignment do not improve by using patient-specific instrumentation in total knee arthroplasty: a randomized controlled trial. Knee Surg Sports Traumatol Arthrosc. [Epub ahead of print] |
| 97 | Kotela A, Lorkowski J, Kucharzewski M, Wilk-Frańczuk M, ͆liwiński Z, Frańczuk B, et al. Patient-specific CT-based instrumentation versus conventional instrumentation in total knee arthroplasty: A prospective randomized controlled study on clinical outcomes and in-hospital data. Biomed Res Int. 2015;2015:165908. |
| 103 | Kwon SK, Yang IH, Bai SJ, Han CD. Periarticular injection with corticosteroid has an additional pain management effect in total knee arthroplasty. Yonsei Medical Journal. 2014;55:493–8. |
| 109 | Liow MHL, Goh GSH, Wong MK, Chin PL, Tay DKJ, Yeo SJ. Robotic-assisted total knee arthroplasty may lead to improvement in quality-of-life measures: a 2-year follow-up of a prospective randomized trial. Knee Surg Sports Traumatol Arthrosc. 2017;25(9):2942–51. |
| 110 | Lizaur-Utrilla A, Sanz-Reig J, Trigueros-Rentero MA. Greater satisfaction in older patients with a mobile-bearing compared with fixed-bearing total knee arthroplasty. J Arthroplasty. 2012;27(2):207–12. |
| 120 | Mayman D, Bourne RB, Rorabeck CH, Vaz M, Kramer J. Resurfacing versus not resurfacing the patella in total knee arthroplasty: 8- to 10-year results. J Arthroplasty. 2003;18:541–5. |
| 123 | Meijerink HJ, Verdonschot N, Van Loon CJM, Hannink G, De Waalmalefijt MC. Similar TKA designs with differences in clinical outcome: A randomized, controlled trial of 77 knees with a mean follow-up of 6 years. Acta Orthop. 2011;82(6):685–91. |
| 129 | Murphy M, Journeaux S, Hides J, Russell T. Does flexion of the femoral implant in total knee arthroplasty increase knee flexion: A randomised controlled trial. Knee. 2014;21(1):257–63. |
| 140 | Park J-W, Kim Y-H. Simultaneous cemented and cementless total knee replacement in the same patients: A prospective comparison of long-term outcomes using an identical design of NexGen prosthesis. Bone Joint J. 2011;93–B(11):1479–86. |
| 143 | Pulavarti RS, Raut V V., McLauchlan GJ. Patella denervation in primary total knee arthroplasty - a randomized controlled trial with 2 years of follow-up. J Arthroplasty. 2014;29(5):977–81. |
| 148 | Roberts DW, Hayes TD, Tate CT, Lesko JP. Selective patellar resurfacing in total knee arthroplasty: A prospective, randomized, double-blind study. J Arthroplasty. 2015;30(2):216–22. |
| 165 | Thomsen MG, Husted H, Bencke J, Curtis D, Holm G, Troelsen A. Do we need a gender-specific total knee replacement?: A randomised controlled trial comparing a high-flex and a gender-specific posterior design. Bone Joint J. 2012;94-B:787-92. |
| 166 | Thomsen MG, Husted H, Otte KS, Holm G, Troelsen A. Do patients care about higher flexion in total knee arthroplasty? A randomized, controlled, double-blinded trial. BMC Musculoskelet Disord. 2013;14:127. |
| 168 | Van Der Ven PJP, Van De Groes S, Zelle J, Koëter S, Hannink G, Verdonschot N. Kneeling and standing up from a chair as performance-based tests to evaluate knee function in the high-flexion range: A randomized controlled trial comparing a conventional and a high-flexion TKA design. BMC Musculoskelet Disord. 2017;18(1). |
| 173 | Wang F, Zhou Y, Sun J, Yang C. Influences of continuous femoral nerve block on knee function and quality of life in patients following total knee arthroplasty. Int J Clin Exp Med. 2015;8(10):19120–5. |
| 174 | Waters TS, Bentley G. Patellar resurfacing in total knee arthroplasty. A prospective, randomized study. J Bone Joint Surg Am. 2003;85–A(2):212–7. |
| 177 | Wylde V, Learmonth I, Potter A, Bettinson K, Lingard E. Patient-reported outcomes after fixed- versus mobile-bearing total knee replacement: A MULTI-CENTRE RANDOMISED CONTROLLED TRIAL USING THE KINEMAX TOTAL KNEE REPLACEMENT. J Bone Joint Surg Br. 2008;90–B(9):1172–9. |
| 178 | Yagishita K, Muneta T, Ju YJ, Morito T, Yamazaki J, Sekiya I. High-flex Posterior Cruciate-Retaining vs Posterior Cruciate-Substituting Designs in Simultaneous Bilateral Total Knee Arthroplasty. A Prospective, Randomized Study. J Arthroplasty. 2012;27(3):368–74. |
| 180 | Zha GC, Sun JY, Dong SJ. Less anterior knee pain with a routine lateral release in total knee arthroplasty without patellar resurfacing: A prospective, randomized study. Knee Surgery, Sport Traumatol Arthrosc [Internet]. 2013/11/30. 2014;22(3):517–25. Available from: http://download.springer.com/static/pdf/386/art%253A10.1007%252Fs00167-013-2789-0.pdf?originUrl=http%3A%2F%2Flink.springer.com%2Farticle%2F10.1007%2Fs00167-013-2789-0&token2=exp=1478596312~acl=%2Fstatic%2Fpdf%2F386%2Fart%25253A10.1007%25252Fs00167-013-278Bjkb |
| **Cohort Studies** | |
| 1 | Adam RF, Noble J. Primary total knee arthroplasty in the elderly. J Arthroplasty. 1994;9(5):495–7. |
| 5 | Ali A, Lindstrand A, Sundberg M, Flivik G. Preoperative Anxiety and Depression Correlate With Dissatisfaction After Total Knee Arthroplasty: A Prospective Longitudinal Cohort Study of 186 Patients, With 4-Year Follow-Up. J Arthroplasty. 2017;32(3):767–70. |
| 6 | Altay MA, C. E, R. A, U.E. I. Midvastus versus medial parapatellar approach in total knee arthroplasty: A comparison of early functional results. Turkiye Klin J Med Sci. 2011;31(5):1106–12. |
| 7 | Anderson JG, Wixson RL, Tsai D, Stulberg SD, Chang RW. Functional outcome and patient satisfaction in total knee patients over the age of 75. J Arthroplasty. 1996;11(7):831–40. |
| 10 | Baker P, Muthumayandi K, Gerrand C, Kleim B, Bettinson K, Deehan D. Influence of Body Mass Index (BMI) on Functional Improvements at 3 Years Following Total Knee Replacement: A Retrospective Cohort Study. PLoS One. 2013;8(3):e59079. |
| 11 | Baker PN, Rushton S, Jameson SS, Reed M, Gregg P, Deehan DJ. Patient satisfaction with total knee replacement cannot be predicted from pre-operative variables alone: A cohort study from the national joint registry for england and wales. Bone Joint J. 2013;95-B(10):1359–65. |
| 12 | Barlow BT, Oi KK, Lee Y yu, Joseph AD, Alexiades MM. Incidence, indications, outcomes, and survivorship of stems in primary total knee arthroplasty. Knee Surg Sports Traumatol Arthrosc. 2017;25(11):3611–9. |
| 14 | Barrack RL, Ruh EL, Chen J, Lombardi A V., Berend KR, Parvizi J, et al. Impact of Socioeconomic Factors on Outcome of Total Knee Arthroplasty. Clin Orthop Relat Res. 2014;472(1):86–97. |
| 16 | Bierke S, Häner M, Petersen W. Influence of somatization and depressive symptoms on the course of pain within the first year after uncomplicated total knee replacement: a prospective study. Int Orthop. 2016;40(7):1353–60. |
| 17 | Bierke S, Petersen W. Influence of anxiety and pain catastrophizing on the course of pain within the first year after uncomplicated total knee replacement: a prospective study. Arch Orthop Trauma Surg. 2017;137(12):1735–42. |
| 18 | Biyani RK, Ziemba-Davis M, Ireland PH, Meneghini RM. Does an Anterior-Lipped Tibial Insert Adequately Substitute for a Post-Cam Articulation in Total Knee Arthroplasty. Surg Technol Int. 2017;30:341–5. |
| 23 | Bugada D, Allegri M, Gemma M, Ambrosoli AL, Gazzerro G, Chiumiento F, et al. Effects of anaesthesia and analgesia on long-term outcome after total knee replacement: A prospective, observational, multicentre study. Eur J Anaesthesiol. 2017;34(10):665-672. |
| 28 | Chang MJ, Kim SH, Kang YG, Chang CB, Kim TK. Activity levels and participation in physical activities by Korean patients following total knee arthroplasty. BMC Musculoskelet Disord. 2014;15(1):240. |
| 30 | Chinnappa J, Chen DB, Harris IA, MacDessi SJ. Predictors and Functional Implications of Change in Leg Length After Total Knee Arthroplasty. 2017;32(9):2725-9. |
| 32 | Choi NY, In Y, Bae JH, Do JH, Chung SJ, Koh IJ. Are Midterm Patient-Reported Outcome Measures Between Rotating-Platform Mobile-Bearing Prosthesis and Medial-Pivot Prosthesis Different? A Minimum of 5-Year Follow-Up Study. J Arthroplasty. 2017;32(3):824–9. |
| 33 | Clement ND, MacDonald D, Burnett R. Primary total knee replacement in patients with mental disability improves their mental health and knee function: A prospective study. Bone Joint J . 2013;95–B(3):360–6 |
| 34 | Clement ND, MacDonald D, Simpson AHRW, Burnett R. Total knee replacement in patients with concomitant back pain results in a worse functional outcome and a lower rate of satisfaction. Bone Joint J. 2013;95 B(12):1632–9. |
| 35 | Clement ND, MacDonald D, Burnett R, Breusch SJ. Diabetes does not influence the early outcome of total knee replacement: A prospective study assessing the Oxford knee score, short form 12, and patient satisfaction. Knee. 2013;20(6):437–41. |
| 36 | Clement ND, Burnett R. Patient satisfaction after total knee arthroplasty is affected by their general physical well-being. Knee Surg Sports Traumatol Arthrosc. 2013;21(11):2638–46. |
| 37 | Clement ND, MacDonald D, Burnett R. Predicting patient satisfaction using the Oxford knee score: Where do we draw the line? Arch Orthop Trauma Surg. 2013;133(5):689–94. |
| 39 | Clement ND, MacDonald D, Burgess AG, Howie CR. Articular surface mounted navigated total knee arthroplasty improves the reliability of component alignment. Knee Surg Sports Traumatol Arthrosc. [Epub ahead of print] |
| 40 | Clement ND, MacDonald D, Patton JT, Burnett R. Post-operative Oxford knee score can be used to indicate whether patient expectations have been achieved after primary total knee arthroplasty. Knee Surg Sports Traumatol Arthrosc. 2015;23(6):1578–90. |
| 41 | Clement ND, Makaram N, Bell J, Tiemessen CH, Mehdi SA, Livingston SJ. Columbus® computer navigated total knee arthroplasty: Gap balancing versus measured resection. Knee. 2017;24(6):1442–7. |
| 42 | Collados-Maestre I, Lizaur-Utrilla A, Martinez-Mendez D, Marco-Gomez L, Lopez-Prats FA. Concomitant low back pain impairs outcomes after primary total knee arthroplasty in patients over 65 years: a prospective, matched cohort study. Arch Orthop Trauma Surg. 2016;136(12):1767–71. |
| 44 | Conditt MA, Noble PC, Bertolusso R, Woody J, Parsley BS. The PCL significantly affects the functional outcome of total knee arthroplasty. J Arthroplasty. 2004;19(7):107–12. |
| 46 | Dixon S, Blom AW, Whitehouse MR, Wylde V. Comparison of patient reported outcomes after Triathlon?? and Kinemax Plus prostheses. Ann R Coll Surg Engl. 2014;96(1):61–6. |
| 49 | Duivenvoorden T, Vissers MM, Verhaar JAN, Busschbach JJ V, Gosens T, Bloem RM, et al. Anxiety and depressive symptoms before and after total hip and knee arthroplasty: A prospective multicentre study. Osteoarthr Cartil. 2013;21(12):1834–40. |
| 50 | Filardo G, Roffi A, Merli G, Marcacci T, Berti Ceroni F, Raboni D, et al. Patients control preferences and results in knee arthroplasty. Knee Surg Sports Traumatol Arthrosc. 2017;25(2):552–8. |
| 51 | Franklin PD, Karbassi J a, Li W, Yang W, Ayers DC. Reduction in narcotic use after primary total knee arthroplasty and association with patient pain relief and satisfaction. J Arthroplasty. 2010;25(6 Suppl):12–6. |
| 53 | Furu M, Ito H, Nishikawa T, Nankaku M, Kuriyama S, Ishikawa M, et al. Quadriceps strength affects patient satisfaction after total knee arthroplasty. J Orthop Sci. 2016;21(1):38–43. |
| 54 | Giurea A, Fraberger G, Kolbitsch P, Lass R, Schneider E, Kubista B, et al. The Impact of Personality Traits on the Outcome of Total Knee Arthroplasty. Biomed Res Int. 2016;2016:5282160. |
| 56 | Goodman SM, Johnson B, Zhang M, Huang W-T, Zhu R, Figgie M, et al. Patients with Rheumatoid Arthritis have Similar Excellent Outcomes after Total Knee Replacement Compared with Patients with Osteoarthritis. J Rheumatol. 2016;43(1):46–53. |
| 57 | Goudie ST, Deakin AH, Ahmad A, Maheshwari R, Picard F. Flexion Contracture Following Primary Total Knee Arthroplasty: Risk Factors and Outcomes. Orthopedics. 2011;34(12):e855-9. |
| 58 | Gustke KA, Golladay GJ, Roche MW, Jerry GJ, Elson LC, Anderson CR. Increased satisfaction after total knee replacement using sensor-guided technology. Bone Joint J. 2014;96B(10):1333–8. |
| 59 | Ha CW, Park YB, Song YS, Kim JH, Park YG. Increased Range of Motion Is Important for Functional Outcome and Satisfaction After Total Knee Arthroplasty in Asian Patients. J Arthroplasty. 2016;31(6):1199–203. |
| 65 | Hinarejos P, Puig-Verdie L, Leal J, Pelfort X, Torres-Claramunt R, Sánchez-Soler J, et al. No differences in functional results and quality of life after single-radius or multiradius TKA. Knee Surg Sports Traumatol Arthrosc. 2016;24(8):2634–40. |
| 66 | Hirschmann MT, Hoffmann M, Krause R, Jenabzadeh RA, Arnold MP, Friederich NF. Anterolateral approach with tibial tubercle osteotomy versus standard medial approah for primary total knee arthroplasty: does it matter? BMC Musculoskelet Disord. 2010;11:167 |
| 68 | Huijbregts HJTAM, Khan RJK, Fick DP, Jarrett OM, Haebich S. Prosthetic alignment after total knee replacement is not associated with dissatisfaction or change in Oxford Knee Score. A multivariable regression analysis. Knee. 2016;23(3):535–9. |
| 70 | Jacobs CA, Christensen CP, Karthikeyan T. Greater Medial Compartment Forces During Total Knee Arthroplasty Associated With Improved Patient Satisfaction and Ability to Navigate Stairs. J Arthroplasty. 2016;31(9):87–90. |
| 73 | Jacobs CA, Christensen CP, Karthikeyan T. Patients with Pain 60 to 120Days after Total Knee Arthroplasty More Likely to be Dissatisfied at Mid-Term Follow-Up. J Arthroplasty. 2015;30(11):1923–6. |
| 74 | Jacobs CA, Christensen CP, Karthikeyan T. An Intact Anterior Cruciate Ligament at the Time of Posterior Cruciate Ligament–Retaining Total Knee Arthroplasty Was Associated With Reduced Patient Satisfaction and Inferior Pain and Stair Function. J Arthroplasty. 2016;31(8):1732–5. |
| 75 | Jain NP, Lee SY, Morey VM, Chong S, Kang YG, Kim TK. Early Clinical Outcomes of a New Posteriorly Stabilized Total Knee Arthroplasty Prosthesis: Comparisons with Two Established Prostheses. Knee Surg Relat Res. 2017;29(3):180-188. |
| 79 | Keurentjes JC, Fiocco M, So-Osman C, Onstenk R, Koopman-Van Gemert AWMM, P??ll RG, et al. Patients with Severe Radiographic Osteoarthritis Have a Better Prognosis in Physical Functioning after Hip and Knee Replacement: A Cohort-Study. PLoS One [Internet]. 2013/04/11. 2013;8(4):e59500. Available from: http://journals.plos.org/plosone/article/asset?id=10.1371/journal.pone.0059500.PDF |
| 80 | Keurentjes JC, Blane D, Bartley M, Keurentjes JJB, Fiocco M, Nelissen RG. Socio-Economic Position Has No Effect on Improvement in Health-Related Quality of Life and Patient Satisfaction in Total Hip and Knee Replacement: A Cohort Study. PLoS One. 2013;8(3):e56785. |
| 81 | Khamis E, Mohammed H, Al Asheeri S, Zainaldeen A, Malki A. Does the implant design influence the outcome after total knee arthroplasty? Bahrain Med Bull. 2015;37(4):246–9. |
| 83 | Kim TK, Cho HJ, Kang YG, Kim SJ, Chang CB. Improved early clinical outcomes of RP/PS mobile-bearing total knee arthroplasties. Clin Orthop Relat Res. 2009;467(11):2901–10. |
| 85 | Kim YH, Park JW. Comparison of highly cross-linked and conventional polyethylene in posterior cruciate-substituting total knee arthroplasty in the same patients. J Bone Joint Surg Am. 2014;96(21):1807–13. |
| 90 | Kim SH, Rhee SM, Lim JW, Lee HJ. The effect of leg length discrepancy on clinical outcome after TKA and identification of possible risk factors. Knee Surg Sports Traumatol Arthrosc. 2015/11/26. 2016;24(8):2678–85. |
| 94 | Klit J, Jacobsen S, Rosenlund S, Sonne-Holm S, Troelsen A. Total knee arthroplasty in younger patients evaluated by alternative outcome measures. J Arthroplasty. 2014;29(5):912–7. |
| 95 | Kornilov N, Lindberg MF, Gay C, Saraev A, Kuliaba T, Rosseland LA, et al. Higher physical activity and lower pain levels before surgery predict non-improvement of knee pain 1 year after TKA. Knee Surg Sports Traumatol Arthrosc. [Epub ahead of print] |
| 99 | Khuangsirikul S, Lekkreusuwan K, Chotanaphuti T. 10-Year patient satisfaction compared between computer-assisted navigation and conventional techniques in minimally invasive surgery total knee arthroplasty. Comput Assist Surg. 2016;21(1):172–5. |
| 101 | Kuroda Y, Matsumoto T, Takayama K, Ishida K, Kuroda R, Kurosaka M. Subjective evaluation before and after total knee arthroplasty using the 2011 Knee Society Score. Knee. 2016;23(6):964–7. |
| 104 | Lehnen K, Giesinger K, Warschkow R, Porter M, Koch E, Kuster MS. Clinical outcome using a ligament referencing technique in CAS versus conventional technique. Knee Surg Sports Traumatol Arthrosc. 2011;19(6):887–92. |
| 105 | Li B, Bai L, Fu Y, Wang G, He M, Wang J. Comparison of clinical outcomes between patellar resurfacing and nonresurfacing in total knee arthroplasty: Retrospective study of 130 cases. J Int Med Res. 2012;40(5):1794–803. |
| 107 | Lingard EA, Sledge CB, Learmonth ID. Patient Expectations Regarding Total Knee Arthroplasty. J Bone Joint Surg. 2006;88(6):1201–7. |
| 111 | Lizaur-Utrilla A, Martinez-Mendez D, Miralles-Muñoz FA, Marco-Gomez L, Lopez-Prats FA. Negative impact of waiting time for primary total knee arthroplasty on satisfaction and patient-reported outcome. Int Orthop. 2016;40(11):2303–7. |
| 112 | Lizaur-Utrilla A, Martinez-Mendez D, Miralles-Muñoz FA, Marco-Gomez L, Lopez-Prats FA. Risk-Benefit on Quality of Life After Total Knee Arthroplasty in Octogenarians. J Arthroplasty. 2017;32(8):2417–20. |
| 115 | Machhindra MV, Kang JY, Kang YG, Chowdhry M, Kim TK. Functional Outcomes of a New Mobile-Bearing Ultra-Congruent TKA System: Comparison With the Posterior Stabilized System. J Arthroplasty. 2015;30(12):2137–42. |
| 116 | Maddali TVP, Sun JY, Zha GC. Outcomes of one-stage versus two-stage total knee arthroplasty for bilateral knee arthritis. Chinese J Tissue Eng Res. 2015;19(9):1321–8. |
| 119 | Matsumoto T, Takayama K, Muratsu H, Ishida K, Hashimoto S, Hayashi S, et al. Relatively Loose Flexion Gap Improves Patient-Reported Clinical Scores in Cruciate-Retaining Total Knee Arthroplasty. J Knee Surg. [Epub ahead of print] |
| 121 | McLawhorn AS, Bjerke-Kroll BT, Blevins JL, Sculco PK, Lee Y yu, Jerabek SA. Patient-Reported Allergies Are Associated With Poorer Patient Satisfaction and Outcomes After Lower Extremity Arthroplasty: A Retrospective Cohort Study. J Arthroplasty. 2015;30(7):1132–6. |
| 122 | Meftah M, White PB, Ranawat AS, Ranawat CS. Long-term results of total knee arthroplasty in young and active patients with posterior stabilized design. Knee. 2016;23(2):318–21. |
| 124 | Meijerink HJ, Brokelman RBG, van Loon CJM, van Kampen A, de Waal Malefijt MC. Surgeon’s expectations do not predict the outcome of a total knee arthroplasty. Arch Orthop Trauma Surg. 2009;129(10):1361–5. |
| 125 | Merle-Vincent F, Couris CM, Schott AM, Conrozier T, Piperno M, Mathieu P, et al. Factors predicting patient satisfaction 2 years after total knee arthroplasty for osteoarthritis. Jointt Bone Spine. 2011;78(4):383–6. |
| 126 | Miner AL, Lingard EA, Wright EA, Sledge CB, Katz JN, Gillespie W, et al. Knee range of motion after total knee arthroplasty: How important is this as an outcome measure? J Arthroplasty. 2003;18(3):286–94. |
| 127 | Mistry D, O’Meeghan C. Fate of the infrapatellar branch of the saphenous nerve post total knee arthroplasty. ANZ J Surg. 2005;75(9):822–4. |
| 128 | Mont MA, Marker DR, Seyler TM, Gordon N, Hungerford DS, Jones LC. Knee arthroplasties have similar results in high- and low-activity patients. Clin Orthop Relat Res. 2007;460(460):165–73. |
| 131 | Nakano N, Matsumoto T, Ishida K, Tsumura N, Kuroda R, Kurosaka M. Long-term subjective outcomes of computer-assisted total knee arthroplasty. Int Orthop. 2013;37(10):1911–5. |
| 132 | Nam D, Nunley RM, Berend KR, Lombardi A V., Barrack RL. The impact of custom cutting guides on patient satisfaction and residual symptoms following total knee arthroplasty. Knee [Internet]. 2016/01/10. 2016;23(1):144–8. Available from: http://ac.els-cdn.com/S096801601500174X/1-s2.0-S096801601500174X-main.pdf?_tid=df0408f0-9c49-11e6-b3ad-00000aacb361&acdnat=1477575357_4238bd0914bbd640e8288f416c112181 |
| 134 | Nam D, Li K, Riegler V, Barrack RL. Patient-Reported Metal Allergy: A Risk Factor for Poor Outcomes After Total Joint Arthroplasty? J Arthroplasty. 2016;31(9):1910–5. |
| 135 | Narayan K, Thomas G, Kumar R. Is extreme flexion of the knee after total knee replacement a prerequisite for patient satisfaction? Int Orthop. 2009;33(3):671–4. |
| 138 | Núñez M, Lozano L, Núñez E, Segur JM, Sastre S, Maculé F, et al. Total knee replacement and health-related quality of life: Factors influencing long-term outcomes. Arthritis Rheum. 2009;61(8):1062–9. |
| 139 | Nunley RM, Nam D, Berend KR, Lombardi A V., Dennis DA, Della Valle CJ, et al. New Total Knee Arthroplasty Designs: Do Young Patients Notice? Clin Orthop Relat Res. 2015;473(1):101–8. |
| 141 | Parsley BS, Conditt MA, Bertolusso R, Noble PC. Posterior Cruciate Ligament Substitution is Not Essential for Excellent Postoperative Outcomes in Total Knee Arthroplasty. J Arthroplasty. 2006;21(6 SUPPL.):127–31. |
| 142 | Pérez-Prieto D, Gil-González S, Pelfort X, Leal-Blanquet J, Puig-Verdié L, Hinarejos P. Influence of depression on total knee arthroplasty outcomes. J Arthroplasty. 2014;29(1):44–7. |
| 144 | Ranawat AS, Rossi R, Loreti I, Rasquinha VJ, Rodriguez JA, Ranawat CS. Comparison of the PFC Sigma Fixed-Bearing and Rotating-Platform Total Knee Arthroplasty in the Same Patient: Short-Term Results. J Arthroplasty. 2004;19(1):35–9. |
| 145 | Ranawat CS, White PB, West S, Ranawat AS. Clinical and Radiographic Results of Attune and PFC Sigma Knee Designs at 2-Year Follow-Up: A Prospective Matched-Pair Analysis. J Arthroplasty. 2017;32(2):431–6. |
| 150 | Schlegel UJ, Bruckner T, Schneider M, Parsch D, Geiger F, Breusch SJ. Surface or full cementation of the tibial component in total knee arthroplasty: a matched-pair analysis of mid- to long-term results. Arch Orthop Trauma Surg. 2015;135(5):703–8. |
| 151 | Schnurr C, Jarrous M, Güdden I, Eysel P, König DP. Pre-operative arthritis severity as a predictor for total knee arthroplasty patients’ satisfaction. Int Orthop. 2013;37(7):1257–61. |
| 152 | Schuster AJ, von Roll AL, Pfluger D, Wyss T. Anteroposterior stability after posterior cruciate-retaining total knee arthroplasty. Knee Surg Sports Traumatol Arthrosc. 2011;19(7):1113–20. |
| 153 | Scott CEH, Howie CR, MacDonald D, Biant LC. Predicting dissatisfaction following total knee replacement: A PROSPECTIVE STUDY OF 1217 PATIENTS. J Bone Joint Surg Br 2010;92–B(9):1253–8. |
| 154 | Scott CEH, Murray RC, MacDonald DJ, Biant LC. Staged bilateral total knee replacement: Changes in expectations and outcomes between the first and second operations. Bone Joint J. 2014;96 B(6):752–8. |
| 155 | Scott CEH, Davidson E, MacDonald DJ, White TO, Keating JF. Total knee arthroplasty following tibial plateau fracture: a matched cohort study. Bone Joint J. 2015;97–B(4):532–8. |
| 156 | Scott CEH, Oliver WM, MacDonald D, Wade FA, Moran M, Breusch SJ. Predicting dissatisfaction following total knee arthroplasty in patients under 55 years of age. Bone Joint J. 2016;98–B(12):1625–34. |
| 157 | Senioris A, Saffarini M, Rahali S, Malekpour L, Dujardin F, Courage O. Does patellofemoral congruence following total knee arthroplasty correlate with pain or function? Intraoperative arthroscopic assessment of 30 cases. Ann Transl Med. 2016;4(15):279–279. |
| 158 | Seo J-G, Moon Y-W, Cho B-C, Kim SC, Ko YH, Jang SP, et al. Is Total Knee Arthroplasty a Viable Treatment Option in Octogenarians with Advanced Osteoarthritis? Knee Surg Relat Res. 2015;27(4):221–7. |
| 160 | Shukla R, Mahajan P, Singh M, Jain R, Kumar R. Outcome of Total Knee Replacement via Two Approaches in Indian Scenario. J Knee Surg. 2016;30(2):174–8. |
| 161 | Singisetti K, Muthumayandi K, Abual-Rub Z, Weir D. Navigation-assisted versus conventional total knee replacement: no difference in patient-reported outcome measures (PROMs) at 1 and 2 years. Arch Orthop Trauma Surg. 2015;135(11):1595–601. |
| 163 | Sun Y, Yang B, Tong S, Sun J, Zhu Y. Patelloplasty Versus Traditional Total Knee Arthroplasty for Osteoarthritis. Orthopedics. 2012;35(3):343–9. |
| 164 | Thambiah MD, Nathan S, Seow BZ, Liang S, Lingaraj K. Patient satisfaction after total knee arthroplasty: An Asian perspective. Singapore Med J. 2015;56(5):259–63. |
| 170 | van Houten AH, Heesterbeek PJC, Wymenga AB. Patella position is not a determinant for anterior knee pain 10 years after balanced gap total knee arthroplasty. Knee Surg Sports Traumatol Arthrosc. 2016;24(8):2656–62. |
| 172 | Von Keudell A, Sodha S, Collins J, Minas T, Fitz W, Gomoll AH. Patient satisfaction after primary total and unicompartmental knee arthroplasty: An age-dependent analysis. Knee. 2014;21(1):180–4. |
| 175 | White PB, Ranawat AS. Patient-Specific Total Knees Demonstrate a Higher Manipulation Rate Compared to “Off-the-Shelf Implants.” J Arthroplasty. 2016;31(1):107–11. |
| **Case-control studies** | |
| 15 | Bican O, Jacovides C, Pulido L, Saunders C, Parvizi J. Total knee arthroplasty in patients with fibromyalgia. J Knee Surg. 2011;24(4):265–71. |
| 20 | Boese CK, Gallo TJ, Plantikow CJ. Range of motion and patient satisfaction with traditional and high-flexion rotating-platform knees. Iowa Orthop J. 2011;31:73–7. |
| 69 | Hwang BH, Yang IH, Han CD. Comparison of patellar retention versus resurfacing in LCS mobile-bearing total knee arthroplasty. Knee Surg Sports Traumatol Arthrosc. 2012;20(3):524–31. |
| 93 | Kim SH, Lee S, Ro DH, Cho Y, Lee YM, Chung KY, et al. Comparison of patellar resurfacing versus preservation in high flexion total knee arthroplasty. Knee Surg Sports Traumatol Arthrosc. 2015;23(6):1782–90. |
| 98 | Krushell RJ, Fingeroth RJ. Primary Total Knee Arthroplasty in Morbidly Obese Patients. A 5- to 14-Year Follow-up Study. J Arthroplasty. 2007;22(6 SUPPL.):77–80. |
| 102 | Kwon SK, Kwon HM, Kong Y, Park KK. The impact of generalized joint laxity on clinical outcomes of total knee arthroplasty. Knee Surg Sports Traumatol Arthrosc. 2017;25(11):3360-3365. |
| 108 | Liow MHL, Goh GSH, Pang HN, Tay DKJ, Lo NN, Yeo SJ. Computer-assisted stereotaxic navigation improves the accuracy of mechanical alignment and component positioning in total knee arthroplasty. Arch Orthop Trauma Surg. 2016;136(8):1173–80. |
| 114 | Lygre SHL, Espehaug B, Havelin LI, Vollset SE, Furnes O. Does patella resurfacing really matter? Pain and function in 972 patients after primary total knee arthroplasty: An observational study from the Norwegian Arthroplasty Register. Acta Orthop. 2010;81(1):99–107. |
| 179 | Yeung E, Jackson M, Sexton S, Walter W, Zicat B. The effect of obesity on the outcome of hip and knee arthroplasty. Int Orthop. 2011;35(6):929–34. |
| **Cross-sectional studies** | |
| 2 | Albayrak I, Apiliogullari S, Erkocak OF, Kavalci H, Ozerbil OM, Levendoglu F. Total Knee Arthroplasty due to Knee Osteoarthritis: Risk Factors for Persistent Postsurgical Pain. J Natl Med Assoc. 2016;108(4):236-243. |
| 3 | Ali A, Sundberg M, Robertsson O, Dahlberg LE, Thorstensson CA, Redlund-Johnell I, et al. Dissatisfied patients after total knee arthroplasty. Acta Orthop. 2014;85(3):229–33. |
| 9 | Baker PN, van der Meulen JH, Lewsey J, Gregg PJ. The role of pain and function in determining patient satisfaction after total knee replacement: DATA FROM THE NATIONAL JOINT REGISTRY FOR ENGLAND AND WALES. J Bone Joint Surg Br 2007;89–B(7):893–900. |
| 21 | Bonnin M, Laurent JR, Parratte S, Zadegan F, Badet R, Bissery A. Can patients really do sport after TKA? Knee Surg Sports Traumatol Arthrosc.. 2010;18(7):853–62. |
| 22 | Bourne RB, Chesworth BM, Davis AM, Mahomed NN, Charron KDJ. Patient satisfaction after total knee arthroplasty: Who is satisfied and who is not? Clin Orthop Relat Res. 2010;468(1):57–63. |
| 24 | Bullens PHJ, Van Loon CJM, De Waal Malefijt MC, Laan RFJM, Veth RPH. Patient satisfaction after total knee arthroplasty: A comparison between subjective and objective outcome assessments. J Arthroplasty. 2001;16(6):740–7. |
| 29 | Chang CB, Yoo JH, Koh IJ, Kang YG, Seong SC, Kim TK. Key factors in determining surgical timing of total knee arthroplasty in osteoarthritic patients: Age, radiographic severity, and symptomatic severity. J Orthop Traumatol. 2010;11(1):21–7. |
| 38 | Clement ND, Jenkins PJ, MacDonald D, Nie YX, Patton JT, Breusch SJ, et al. Socioeconomic status affects the Oxford knee score and Short-Form 12 score following total knee replacement. Bone Joint J.2013;95–B(1):52–8. |
| 45 | Devers BN, Conditt MA, Jamieson ML, Driscoll MD, Noble PC, Parsley BS. Does Greater Knee Flexion Increase Patient Function and Satisfaction After Total Knee Arthroplasty? J Arthroplasty. 2011;26(2):178–86. |
| 47 | Dhurve K, Scholes C, El-Tawil S, Shaikh A, Weng LK, Levin K, et al. Multifactorial analysis of dissatisfaction after primary total knee replacement. Knee. 2017;24(4):856–62. |
| 48 | Dickstein R, Heffes Y, Shabtai EI, Markowitz E. Total knee arthroplasty in the elderly: patients’ self-appraisal 6 and 12 months postoperatively. Gerontology. 1998;44(4):204-10. |
| 55 | Gong L, Dong JY. Patient’s personality predicts recovery after total knee arthroplasty: A retrospective study. J Orthop Sci. 2014;19(2):263–9. |
| 62 | Hawker G, Wright J, Coyte P, Paul J, Dittus R, Croxford R, et al. Health-related quality of life after knee replacement. J Bone Joint Surg Am. 1998;80(2):163–73. |
| 63 | Heesterbeek PJC, van Houten AH, Klenk JS, Eijer H, Christen B, Wymenga AB, et al. Superior long-term survival for fixed bearing compared with mobile bearing in ligament-balanced total knee arthroplasty. Knee Surg Sports Traumatol Arthrosc. [Epub ahead of print] |
| 71 | Jacobs CA, Christensen CP, Karthikeyan T. Patient and Intraoperative Factors Influencing Satisfaction Two to Five Years After Primary Total Knee Arthroplasty. J Arthroplasty. 2014;29(8):1576–9. |
| 72 | Jacobs CA, Christensen CP. Factors influencing patient satisfaction two to five years after primary total knee arthroplasty. J Arthroplasty. 2014;29(6):1189–91. |
| 77 | Kawahara S, Okazaki K, Matsuda S, Nakahara H, Okamoto S, Iwamoto Y. Internal rotation of femoral component affects functional activities after TKA-survey with the 2011 knee society score. J Arthroplasty. 2014;29(12):2319–23. |
| 86 | Kim TK, Chang CB, Kang YG, Kim SJ, Seong SC. Causes and Predictors of Patient’s Dissatisfaction After Uncomplicated Total Knee Arthroplasty. J Arthroplasty. 2009;24(2):263–71. |
| 89 | Kim TK, Kwon SK, Kang YG, Chang CB, Seong SC. Functional disabilities and satisfaction after total knee arthroplasty in female asian patients. J Arthroplasty. 2010;25(3):452–8. |
| 100 | Kuriyama S, Ishikawa M, Nakamura S, Furu M, Ito H, Matsuda S. Noise Generation With Good Range of Motion but Without Femorotibial Instability Has Small Effect on Patient Satisfaction After Total Knee Arthroplasty. J Arthroplasty. 2017;32(2):407–12. |
| 106 | Lim JBT, Loh B, Chong HC, Tan AHC. History of previous Knee Surg does not affect the clinical outcomes of primary total knee arthroplasty in an Asian population. Ann Transl Med. 2016;4(16):303–303. |
| 113 | Losina E, Plerhoples T, Fossel AH, Mahomed NN, Barrett J, Creel AH, et al. Offering patients the opportunity to choose their hospital for total knee replacement: Impact on satisfaction with the surgery. Arthritis Care Res. 2005;53(5):646–52. |
| 117 | Mannion AF, Kämpfen S, Munzinger U, Kramers-de Quervain I. The role of patient expectations in predicting outcome after total knee arthroplasty. Arthritis Res Ther. 2009;11(5):R139. |
| 118 | Matsuda S, Kawahara S, Okazaki K, Tashiro Y, Iwamoto Y. Postoperative alignment and ROM affect patient satisfaction after TKA knee. Clin Orthop Relat Res. 2013;471(1):127–33. |
| 130 | Nakahara H, Okazaki K, Mizu-uchi H, Hamai S, Tashiro Y, Matsuda S, et al. Correlations between patient satisfaction and ability to perform daily activities after total knee arthroplasty: why aren’t patients satisfied? J Orthop Sci. 2015;20(1):87–92. |
| 133 | Nam D, Nunley RM, Barrack RL. Patient dissatisfaction following total knee replacement: a growing concern? Bone Joint J. 2014;96–B(11_Supple_A):96–100. |
| 136 | Nishio Y, Onodera T, Kasahara Y, Takahashi D, Iwasaki N, Majima T. Intraoperative Medial Pivot Affects Deep Knee Flexion Angle and Patient-Reported Outcomes After Total Knee Arthroplasty. J Arthroplasty. 2014;29(4):702–6. |
| 137 | Noble PC, Conditt MA, Cook KF, Mathis KB. The John Insall Award: Patient expectations affect satisfaction with total knee arthroplasty. Clin Orthop Relat Res. 2006;452(452):35–43. |
| 146 | Razmjou H, Boljanovic D, Wright S, Murnaghan J, Holtby R. Association between neuropathic pain and reported disability after total knee arthroplasty. Physiother Canada. 2015;67(4):311–8. |
| 147 | Roberts VI, Esler CNA, Harper WM. A 15-year follow-up study of 4606 primary total knee replacements. J Bone Joint Surg Br 2007;89–B(11):1452–6. |
| 149 | Robertsson O, Dunbar M, Pehrsson T, Knutson K, Lidgren L. Patient satisfaction after knee arthroplasty: A report on 27,372 knees operated on between 1981 and 1995 in Sweden. Acta Orthop Scand. 2000;71(3):262–7. |
| 159 | Sharkey PF, Miller AJ. Noise, Numbness, and Kneeling Difficulties After Total Knee Arthroplasty. Is the Outcome Affected? J Arthroplasty. 2011;26(8):1427–31. |
| 162 | Stickles B, Phillips L, Brox WT, Owens B, Lanzer WL. Defining the relationship between obesity and total joint arthroplasty. Obes Res. 2001;9(3):219–23. |
| 167 | Tsukiyama H, Kuriyama S, Kobayashi M, Nakamura S, Furu M, Ito H, et al. Medial rather than lateral knee instability correlates with inferior patient satisfaction and knee function after total knee arthroplasty. Knee. 2017;24(6):1478–84. |
| 169 | van de Groes SAW, Koëter S, de Waal Malefijt M, Verdonschot N. Effect of medial-lateral malpositioning of the femoral component in total knee arthroplasty on anterior knee pain at greater than 8years of follow-up. Knee. 2014;21(6):1258–62 |
| 171 | Vissers MM, De Groot IB, Reijman M, Bussmann JB, Stam HJ, Verhaar JAN. Functional capacity and actual daily activity do not contribute to patient satisfaction after total knee arthroplasty. BMC Musculoskelet Disord. 2010;11:121. |
| 176 | Williams DP, O’Brien S, Doran E, Price AJ, Beard DJ, Murray DW, et al. Early postoperative predictors of satisfaction following total knee arthroplasty. Knee. 2013;20(6):442–6. |
| **Case-series studies** | |
| 76 | Kaneko T, Kono N, Mochizuki Y, Hada M, Toyoda S, Musha Y. Bi-cruciate substituting total knee arthroplasty improved medio-lateral instability in mid-flexion range. J Orthop. 2017;14(1):201–6. |
| 181 | Zha GC, Feng S, Chen XY, Guo KJ. Does the grading of chondromalacia patellae influence anterior knee pain following total knee arthroplasty without patellar resurfacing? Int Orthop. [Epub ahead of print] |
