# Supplementary material for: Why are patients dissatisfied following a total knee replacement? A systematic review
Source: Int Orthop. 2020 Jul 8;44(10):1971–2007. doi: 10.1007/s00264-020-04607-9 (PMC7584563; doi:10.1007/s00264-020-04607-9)
Supplement: Supplementary file 3 — (DOCX 17.8 kb) [file 264_2020_4607_MOESM3_ESM.docx]

**Appendix 3**

*1. Patient Demographics (Figure 3a):*

Young age was reported as a factor for satisfaction in four studies and older age was reported as a factor for satisfaction in four studies as well. However, nine studies　reported that age does not act as a factor in influencing patient satisfaction. The second factor is gender where four studies reported that being a male influenced satisfaction and one study reported being a female does the same. But five studies considered gender as not a factor affecting satisfaction. Normal BMI was considered to be a factor for satisfaction in five studies, and seven studies reported it does not have a role in patient satisfaction. One study reported that being Caucasian is a factor for patient satisfaction and one study did not support this. Annual income of more than 25,000 USD was also reported to lead to satisfaction. Finally, three studies reported that social background (education, employment, and insurance) does not affect patient satisfaction while one study supported higher education to be a factor influencing satisfaction.

*2. Non-knee factors (Figure 3b):*

Lack of low back pain, allergies, fibromyalgia**,** other joint problems, use of narcotics**,** medical comorbidities, and ASA (American Society of Anaesthesiologist) grade 2 or worse were all found to be factors that related to patient satisfaction. However, diabetes mellitus**,** generalised joint laxity and pre-operative activity level were not found to affect patient satisfaction. Thirteen studies in total supported that a negative history of mental health problems was a factor related to patient satisfaction compared with 2 studies, which reported no correlation.

*3. Knee factors (Figure 3c):*

Chronic pain, lack of a stiff knee, movement elicited pain, no pain at rest, intact anterior cruciate ligament, knee extension strength, greater intra-operative force in the medial compartment, medial pivot kinematic pattern, high flexion activities and severe pre-operative radiographic degenerative change were reported to be factors related to satisfaction. Four factors were reported not to impact patient satisfaction including history of previous knee surgeries, satisfaction on the first side, chondromalacia patellae, and patellar congruence. Patients with rheumatoid arthritis (RA) were reported to be more satisfied than those with OA, and non-OA diagnosis as a reason for TKR was also considered to be a factor for satisfaction. One study showed patients with RA were more satisfied, followed by OA, post-trauma, and avascular necrosis (AVN). Four studies, however, reported that diagnosis was not a factor related to patient satisfaction.

*4. Factors related to Implant/Prosthesis (Figure 3d):*

High flexion design, gender specific design, material of femoral component, highly cross-linked polyethylene and stem type, number and use were reported as not being factors related to patient satisfaction. Some studies showed that using specific prosthesis can be a factor for patient satisfaction such as Triathlon over Kinemax, PFC over CKS, Vega, Genesis II over E.motion and NexGen over AGC. But two studies reported that the use of specific prosthesis does not affect patient satisfaction. Single radius design was shown to relate to patient satisfaction in one study, and was not considered a factor in another study**.** In addition, posterior stabilised design was reported to be a factor in one study and not a factor in seven studies. The design of the bearing was also found to play a role in patient satisfaction. Patients with mobile bearing insert tended to be more satisfied than those with fixed bearing insert. With regard to the type of mobile bearing, rotating mobile design was reported to be better than floating mobile design. However, seven studies reported that the design of bearing has no impact on patient satisfaction. Use of non-customised prosthesis was reported to be better for satisfaction than use of customised one, and this was not opposed in any other study.

*5. Intra-operative technical factors (Figure 3e):*

Kinematic alignment technique, lateral retinacular release, minimally invasive surgery, periarticular injection with corticosteroid, use of a tourniquet, removal of fat pad**,** one stage/two stage bilateral TKR and gap balancing/measured resection technique were reported not to affect patient satisfaction. Patellar denervation and patelloplasty were reported to be factors affecting satisfaction without being opposed by any other study. Lateral subvastus approach was reported to be a factor affecting satisfaction in one study and three studies refuted the relationship between approach/incision and satisfaction. Using a navigation system was also reported to be a factor affecting satisfaction in one study and not a factor in 12 studies. Surface cementing of the tibial component was shown to be a factor for satisfaction over the full-cementing technique in one study and cementing technique had no effect on patient satisfaction in three studies. Finally, patellar resurfacing was considered a factor affecting satisfaction in four studies and the opposite view in nine studies.

*6. Post-operative outcome factors (Figure 3f):*

Good post-operative alignment, good physical function, no or less pain, no neuropathic pain, no flexion contracture, no knee swelling, no perception of leg length discrepancy, no residual symptoms, no medial joint laxity, no deep prosthetic infection, no complications, pre-operative expectations met and good ligament balance of the knee were found to be factors affecting patient satisfaction without any study opposing them. Five factors were found not to have any role in affecting patient satisfaction without any studies opposing them, which were lateral joint laxity, noise, anterior-posterior knee stability, malpositioning of the tibial component and radiological leg length discrepancy. Improvement in ROM post-operatively was reported to be a factor affecting satisfaction in six studies but three studies reported that this does not affect patient satisfaction. Regarding positioning of the femoral component, accurate coronal alignment, accurate rotation and medial malpositioned component were reported to be factors affecting patient satisfaction while one study reported that positioning of femoral component does not affect patient satisfaction. One study reported that patients without post-operative numbness were more satisfied than those with it but one study refuted this claim.

*7. Surgeon and healthcare factors (Figure 3g):*

Short hospital stay was considered to be a factor affecting satisfaction in one study and it was refuted in another. Type of analgesia**,** country where TKR was performed, surgeon’s job title and surgeon’s satisfaction were considered to have no effect on patient satisfaction. However, high patient motivation for rehabilitation, regular physical activity, waiting time shorter than six months, choice of hospital and continuous irrigation by cold saline and epinephrine were reported to be factors affecting satisfaction.
